# Are phylogenetic trees suitable for chemogenomics analyses of bioactivity data sets: the importance of shared active compounds and choosing a suitable data embedding method, as exemplified on Kinases

**DOI:** 10.1186/1758-2946-5-49

**Published:** 2013-12-13

**Authors:** Shardul Paricharak, Tom Klenka, Martin Augustin, Umesh A Patel, Andreas Bender

**Affiliations:** 1 Unilever Centre for Molecular Science Informatics, Department of Chemistry, University of Cambridge, Lensfield Road, CB2 1EW Cambridge, UK; 2 Merck Millipore, Dundee Technology Park, DD2 1SW Dundee, UK; 3 EMD Millipore Corporation, 15 Research Park Drive, St. Charles, Missouri 63304, USA

**Keywords:** Kinase inhibitor, Selectivity, Phylogenetics, Chemogenomics, Polypharmacology

## Abstract

**Background:**

‘Phylogenetic trees’ are commonly used for the analysis of chemogenomics datasets and to relate protein targets to each other, based on the (shared) bioactivities of their ligands. However, no real assessment as to the suitability of this representation has been performed yet in this area. We aimed to address this shortcoming in the current work, as exemplified by a kinase data set, given the importance of kinases in many diseases as well as the availability of large-scale datasets for analysis. In this work, we analyzed a dataset comprising 157 compounds, which have been tested at concentrations of 1 μM and 10 μM against a panel of 225 human protein kinases in full-matrix experiments, aiming to explain kinase promiscuity and selectivity against inhibitors. Compounds were described by chemical features, which were used to represent kinases (*i.e.* each kinase had an active set of features and an inactive set).

**Results:**

Using this representation, a bioactivity-based classification was made of the kinome, which partially resembles previous sequence-based classifications, where particularly kinases from the TK, CDK, CLK and AGC branches cluster together. However, we were also able to show that in approximately 57% of cases, on average 6 kinase inhibitors exhibit activity against kinases which are located at a large distance in the sequence-based classification (at a relative distance of 0.6 – 0.8 on a scale from 0 to 1), but are correctly located closer to each other in our bioactivity-based tree (distance 0 – 0.4). Despite this improvement on sequence-based classification, also the bioactivity-based classification needed further attention: for approximately 80% of all analyzed kinases, kinases classified as neighbors according to the bioactivity-based classification also show high SAR similarity (*i.e.* a high fraction of shared active compounds and therefore, interaction with similar inhibitors). However, in the remaining ~20% of cases a clear relationship between kinase bioactivity profile similarity and shared active compounds *could not* be established, which is in agreement with previously published atypical SAR (such as for LCK, FGFR1, AKT2, DAPK1, TGFR1, MK12 and AKT1).

**Conclusions:**

In this work we were hence able to show that (1) targets (here kinases) with few shared activities are difficult to establish neighborhood relationships for, and (2) phylogenetic tree representations make implicit assumptions (*i.e.* that neighboring kinases exhibit similar interaction profiles with inhibitors) that are not always suitable for analyses of bioactivity space. While both points have been implicitly alluded to before, this is to the information of the authors the first study that explores both points on a comprehensive basis. Excluding kinases with few shared activities improved the situation greatly (the percentage of kinases for which no neighborhood relationship could be established dropped from 20% to only 4%). We can conclude that all of the above findings need to be taken into account when performing chemogenomics analyses, also for other target classes.

## Background

**Figure 1:**
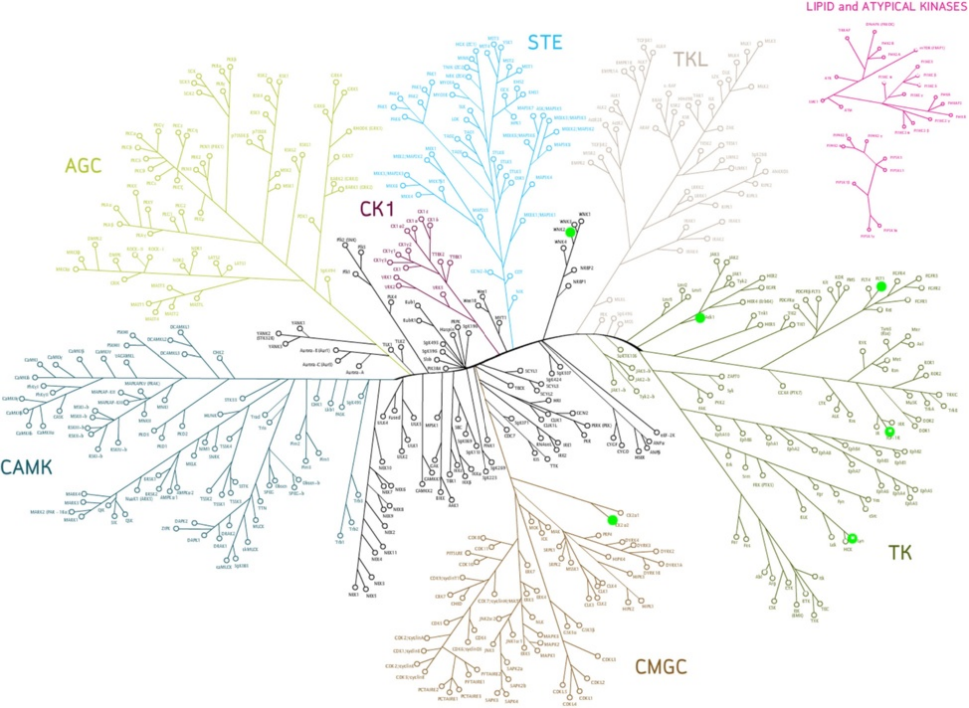
**The human kinome as described by Manning *et al. *[**[5]**] on the basis of sequence similarity.** Outlier kinases (marked in green) will be discussed later in the text. In our revised analysis, kinases showed much better agreement with respect to the expected negative relationship between SAC score (a score based on the fraction of shared active compounds between kinases) and bioactivity distance: only 7 kinases (VEGFR3, ACK1, LYN, CSK21, CSK22, IGF1R and WNK2) were classified as outliers. CSK21 and CSK22 are represented by the same kinase in the tree above and therefore, there are only 6 distinct kinases marked.

Protein kinases are an important class of proteins which are involved in various essential cellular functions, including signaling, growth, development and homeostasis [1-3]. They exert their regulatory effects by phosphorylating serine, threonine or tyrosine residues on substrates which in turn regulates protein activity, localization and function. This is achieved by inducing conformational changes in the substrate protein, leading to events such as the activation of signaling cascades [1]. Counteracting kinases are phosphatases, which generally lead to deactivation of a phosphorylated protein [4], and for normal development of a cell (and hence a healthy state of the organism) fine-tuning of phosphorylation and dephosphorylation processes is of crucial importance [4]. The human kinome contains an estimated 518 protein kinases (Figure 1), as determined by sequence analysis of the human genome via a Hidden Markov Model (HMM) [5,6].

Deregulations of kinases as a consequence of mutations as well as over- or underexpression can cause abnormal changes in cellular signaling and, as a consequence, have implications for the progression of diseases such as cancer, diabetes and inflammation [1]. In case of cancer, the kinases involved are often over-activated, generally leading to excessive cell proliferation (and decreased response to apoptotic signals). Therefore, kinases are common targets of anti-cancer drugs and cancer treatment by kinase inhibition has been one of the most active areas across the pharmaceutical industry in the last decade [1,7]. An example of a highly successful drug in this area is the kinase inhibitor Gleevec which was first developed as a selective Bcr-Abl inhibitor for treatment of chronic myelogenous leukemia (CML) [8-10]. Later, also its secondary activities against KIT were exploited for treating gastrointestinal stromal tumors [11]. Other anti-cancer drugs that were the result of approvals in the last decade include Iressa (indicated in case of EGFR upregulation) [12] and Tykerb (indicated in case of ERBB2 overexpression) [13].

While kinases are involved in various signaling processes, they are still structurally very similar when it comes to their ATP binding sites, which are highly conserved [14,15]. Despite the success of type I kinase inhibitors on the market (*i.e.* inhibitors that target the ATP binding site), virtually no kinase inhibitor is truly selective (although this promiscuity might very well be tolerated in the clinic) [16]. Whilst the promiscuity of kinase inhibitors may hence not necessarily be a problem and may even be beneficial in some cases (such as in case of repurposing Gleevec as described above), it is generally important to understand the inhibition profile of kinase inhibitors early on in the drug discovery process in order to be able to assess efficacy, off-target effects and to anticipate possible safety problems [17-20].

In an attempt to understand the inhibition profile of kinase inhibitors and drug candidates in general, various chemogenomics methods have been employed to analyze compound activity against a series of targets in recent years [21-29]. Many of those studies have indicated that sequence similarity between kinases does *not always* correlate with kinase inhibitor interaction (*i.e.* kinases with dissimilar sequences can also bind to the same compound). One such example is a study by Karaman *et al.*, where the bioactivity profiles of 38 kinase inhibitors tested against 317 kinases was analyzed. The authors found that for the 317 kinases analyzed, compounds originally described as tyrosine kinase inhibitors indeed bound tyrosine kinases more frequently than serine/threonine kinases; however many of the serine/threonine kinase inhibitors were found to interact with tyrosine kinases more frequently [30]. Fabian *et al.* showed that BIRB-796 was able to bind the serine-threonine kinase p38, and the tyrosine kinase ABL(T315I) rather tightly (at around 40 nM), despite both kinases having only a 23% sequence identity [3]. Similarly, the tyrosine kinase inhibitor dasatinib [31] also interacts with serine/threonine kinases, albeit with a 2.9-fold lower selectivity at a concentration of 3 μM than for tyrosine kinases (*i.e.* dasatinib bound to 2.9 times as many tyrosine kinases as it did to serine/threonine kinases) [30]. Also surprising cases of relative selectivity exist, however: while imatinib inhibits LCK, it is selective over the closely related kinase SRC, as shown in the analysis by Fabian *et al*[3].

While above methods did not consider the spatial structure of the ligand binding pocket, also structure-based studies have been performed on kinases, such as by Kuhn *et al.*[32] as well as others [33]. The approach by Kuhn *et al.*, which incorporates the comparison of 3D binding site descriptors across kinases via Cavbase, has shown that kinase binding site properties can be used to predict kinase interaction with inhibitors, such as the cross-reactivity of Gleevec. The model showed separation of serine/threonine and tyrosine kinases and a clustering on the subfamily level could be achieved, where 12 out of the 16 subfamily clusters formed included at most one member from another kinase class. Moreover, the sequence-based similarity of kinases was compared to their Cavbase similarity: in many cases kinase pairs exhibit a sequence identity below 50%, while possessing a Cavbase R_1_ similarity score of 22 or above (*i.e.* high predicted SAR similarity, where SAR similarity specifically refers to *similarity in terms of the compounds target proteins bind to*). Also in the area of predictive modeling, Martin *et al.*[34] developed Bayesian QSAR models on 92 kinases that were diverse in terms of sequence, covering most of kinase sequence space. Subsequently, activities of compounds on previously untested kinases could be predicted as a weighted average of prediction of the same compounds from neighboring models, allowing for assessment of compound promiscuity within the kinome [34]. Whilst this approach has generally proven useful for prediction of bioactivity profiles (an R^2^ value of 0.48 was obtained when tested on validation data from 18 assays) [34], the assumption that kinases that are similar in terms of protein sequence have a similar interaction profile with inhibitors has not been verified thoroughly in this previous work (which is one of the foci of the current work).

As an extension of the work mentioned above and complementary to sequence-based analysis of kinases, Bamborough *et al.* analyzed kinase bioactivity data based on inhibitor affinity fingerprints, and used this approach to rationalize cross-reactivity of compounds [21]. The kinome tree was reclassified using affinity fingerprints, and the relationship between domain sequence identity and kinase SAR similarity was analyzed. The main finding was that there was no linear relationship between kinase sequence similarity and SAR similarity. However, two groups of distinct kinase-pair relationships were observed: pairs of kinases with below 40-50% sequence identity in their kinase domains were found to exhibit significantly lower SAR similarity than kinase pairs with more than 40-50% sequence identity. A similar analysis was performed on another kinase panel by Davis *et al.*[35] where selectivity scores were computed for each kinase by dividing the number of compounds bound with K_d_ < 3 μM by the total number of compounds screened. The results primarily illustrated kinase promiscuity: 60% of the kinases interacted with 10-40% of the compounds and most compounds had interactions with kinases from multiple groups, which was in line with the analysis by Bamborough *et al.*[21].

We will now outline how the current study extends previous approaches. In both the preceding analyses, binary affinity fingerprints were used; *i.e.* inhibitors were classified as either ‘active’ or ‘inactive’. In this work, we extend that approach by incorporating the analysis of chemical features of the inhibitors (*i.e.* by classifying inhibitors as a collection of chemical features), which considerably enhances the statistical power of models (since there are many more features that can be matched than entire compounds). Kinase-pair distance were calculated based on the presence and absence of these chemical features in active and inactive inhibitors, hereby adding more chemical information to the dataset for better comparison of inhibitor cross-reactivity (actual percentage inhibition values were not used for this purpose, because in this case it would not be possible to incorporate the information of chemical features). We set out to analyze a dataset of 157 kinase inhibitors, selected on basis of structural diversity, cell permeability, reversibility and potency [36] and assayed at concentrations of 1 μM and 10 μM against a panel of 225 human protein kinases (this dataset has been made publicly available via ChEMBL recently) [37]. The classification of the kinome was revised, based on bioactivity data and chemical feature enrichments with the aim to rationalize (and predict) cross-reactivity of compounds within the kinome. We show that this classification will more accurately define kinase neighbors in terms of bioactivity similarity in response to inhibitors, and will therefore be more valuable in predicting kinase inhibitor promiscuity. In particular, we will analyze the influence of data density on chemogenomics analyses (which was found to be very important, to the extent that part of the data effectively needs to be removed), as well as revisit the assumptions that phylogenetic trees make when representing similarities between proteins according to ligand similarity (where the assumption that close neighbors exhibit similar compound interaction is invalid in some cases).

## Results and discussion

### Bioactivity dataset

We firstly aimed to understand the nature of our dataset by analyzing physicochemical property diversity and scaffold diversity. The chemical diversity of the kinase inhibitor library analyzed here, compared to 11,577 protein kinase inhibitors retrieved from ChEMBL exhibiting IC_50_ values lower than 10 μM, is shown in Additional file 1: Figure S1 with diverse structures being visualized. PC1 (principal component 1) and PC2 (principal component 2) capture 46% of all variance in the dataset and are related to molecular size (PC1) and charge and lipophilicity (PC2). The Calbiochem library used in the current study covers the left hand side of the PCA space (representing smaller compounds) rather well, whereas the right hand side (representing larger compounds) is not covered as well. The frequency of the top 10 most prevalent scaffolds in the inhibitors is shown in Additional file 2: Figure S2. Given that there were over 110 scaffolds present in a dataset with only 157 inhibitors, we consider this dataset to be highly diverse, which was also one of its original design principles.

**Figure 2:**
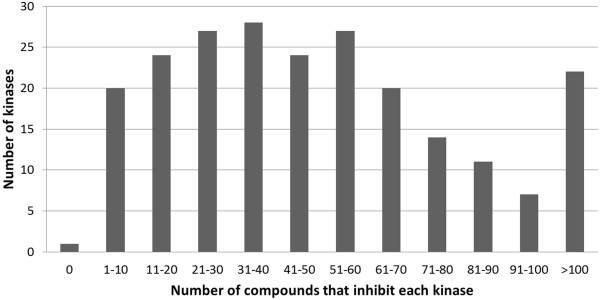
**Distribution of the number of kinases as a function of compounds inhibiting the respective number of kinases.** Compounds that lowered bioactivity of the kinase in question by 50% or more were considered active. Overall, kinases in the dataset were inhibited by 1 to 70 compounds in most cases. This indicates that our kinome dataset contains a wide range of kinases, including those that are promiscuous to multiple compounds as well as selective kinases.

**Figure 3:**
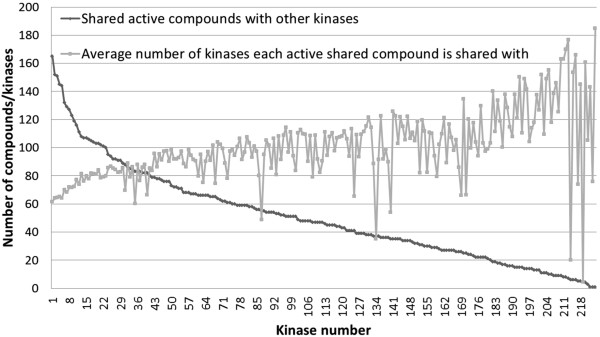
**Compound promiscuity.** The number of shared active compounds with other (1 or more) kinases is shown in dark gray, whereas the average number of kinases these active compounds are shared with for each kinase is shown in light gray. Only 1 kinase (NEK7) does not share any active compounds with other kinases. Of all kinases, 80% share over 20 active compounds which is of importance for establishing meaningful kinase SAR relationships. These compounds are shared with 51 kinases on average, suggesting the compouunds in the dataset to be relatively promiscuous on average.

The bioactivity matrix of 157 compounds against 225 kinases is shown in Additional file 3: Figure S3 and given the importance of the data structure and density (as we will see later) this will be discussed here in some detail. This dataset very much resembles the slightly larger dataset (178 compounds) analyzed by Anastassiadis *et al.*[38], which contains 88% of the compounds used in our dataset. Of all data present in the dataset, 16.1% of all compound-target interactions represent inhibition by at least 50% (which makes this a relatively ‘dense’ dataset, compared to hit rates in typical HTS campaigns) and only 2% represent inhibition between 40% and 60% (the exact distribution is shown in Additional file 4: Figure S4). Hence, the loss of information involved when using a binary cut-off for the classification of active and inactive compounds is minimal [39]. On average, the compounds inhibited 39 kinases, with four structures inhibiting more than 183 kinases (μ + 3SD), namely the known pan-kinase inhibitor Staurosporine (at 1 μM and 10 μM), a compound primarily annotated as a Cdk1/2 inhibitor, the structure K-252a and a PKR inhibitor (10 μM). Overall, kinases in the dataset showed a large variation in their associated number of inhibitors: 76% of kinases were inhibited by 10 to 70 compounds, only a single kinase (NEK7) was not inhibited by any compound, and the remaining kinases were inhibited by 71 or more compounds (for a visualization see Figure 2). This indicates that our kinome dataset contains both kinases that are promiscuous to multiple compounds (*e.g.* > 40) as well as selective kinases (*e.g.* < 20). Furthermore, 180 kinases (80% of all kinases) share at least 20 activities with other kinases, with the average number of shared activities being 51 (+/- 34). The average number of kinases with which active compounds were shared was 101 (+/- 25, corresponding to 45% of all kinases). The distribution for shared activities both in terms of the number of compounds shared (same compounds at different concentrations are considered unique in this case), as well as the number of kinases these compounds are shared with, is shown for each kinase in Figure 3. As mentioned earlier, only a single kinase, namely NEK7, was not inhibited by any compound, and therefore did not share any active compounds either. These data suggest that the compounds in the dataset overall show sufficient shared activities between kinases (and large enough a number of active data points in the first place). While we will discuss later that this was overall indeed found to be true, we will also show the limitations of this statement in detail later in this Results and discussion section.

### Bioactivity-based classification of kinases compared to earlier classifications

**Figure 4:**
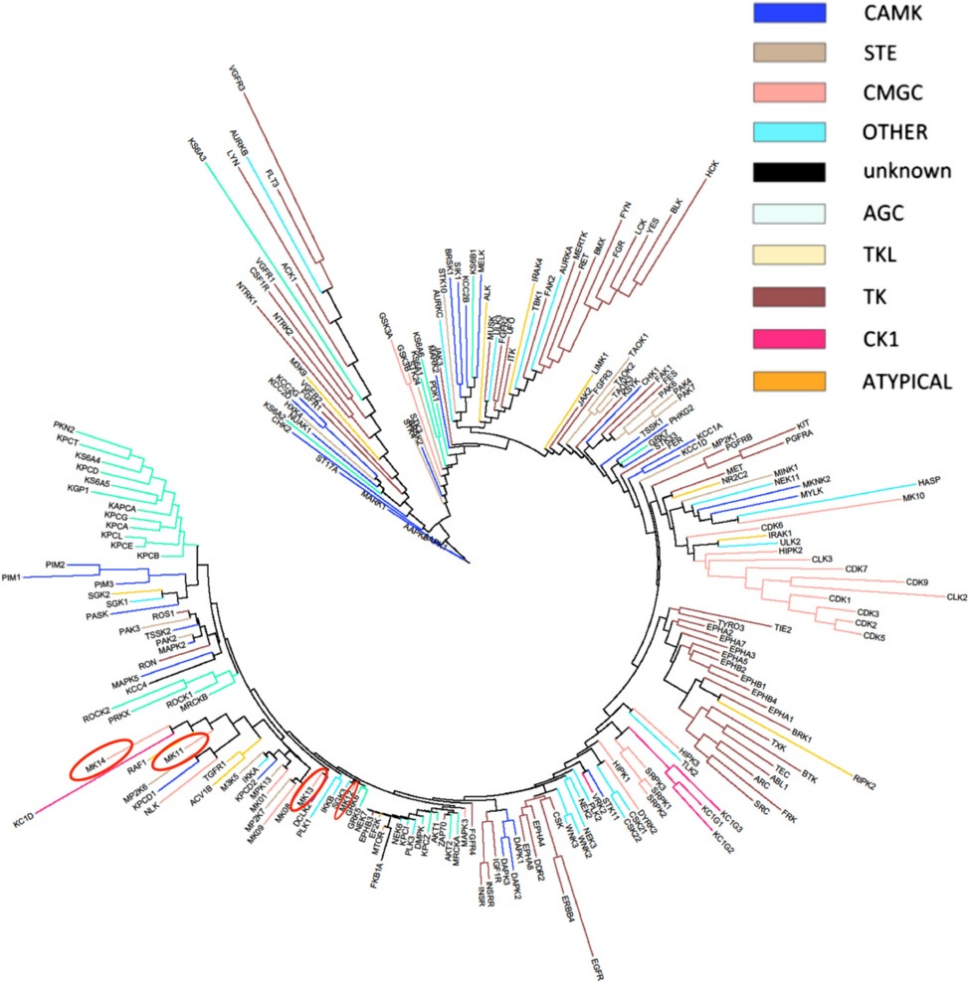
**Kinome tree constructed with a distance matrix based on fingerprint enrichments.** Kinases were colored based on the classification of kinase groups as defined by Manning *et al.*[5] Overall, kinases from the same group (with the same color) tend to group in the same cluster. CDK and CLK kinases from the CMGC group and AGC kinases are clearly grouped together. However, kinases from the same family or group do not always cluster together, as for example is the case with MK12 (p38γ) and MK13 (p38δ), which are both isolated from each other. On the other hand, the very similar proteins MK14 (p38α) and MK11 (p38β) are located close to each other.

The phylogenetic tree generated from the bioactivity matrix (for details see Methods section) is shown in Figure 4. The revised classification of the kinome tree (limited to the kinases in our dataset), based on bioactivity profiles, is overall in good agreement with the sequence-based kinase classification by Manning *et al.*[5], where a Hidden Markov Model of the eukaryotic protein kinase domain was first used to scan the human proteome for kinases, after which sequence alignment between kinases was extended to full-length gene predictions using a combination of EST and cDNA data. Generally, kinases from the same phylogenetic group as defined by Manning *et al.* tend to group in the same cluster in our revised tree based on bioactivity profiles. CDK and CLK kinases from the CMGC group are grouped together, as are the protein C kinases from the AGC group (*e.g.* KPCA and KPCB). Tyrosine kinases also tend to cluster together, of which particularly the Ephrin kinases do so: only 14% of the tyrosine kinases in the dataset were not placed near other tyrosine kinases, compared to 27% of CMGC and 29% of AGC kinases. These findings are in accordance with the analysis by Bamborough *et al.*[21], where Ephrin kinases, and kinases in the TK, AGC and CMGC branches tend to group together. However, kinases from the same family or group do not always cluster, as for example is the case with MK12 (p38γ) and MK13 (p38δ), which are both at a large distance from each other in the bioactivity-based phylogenetic tree. On the other hand, the very similar proteins MK14 (p38α) and MK11 (p38β) are located close to each other. This pattern has also been described earlier in the analysis by Bamborough *et al.*[21], where the difference in activity of MK12 was explained by the presence of a different gatekeeper in its active site as compared to MK14 and MK11.

In addition, we examined an earlier evaluation of interaction maps of 37 known kinase inhibitors [30] against a panel of 317 kinases in more detail. For six of the existing inhibitors (Gefitinib, SU-14813, BIRB-796, Staurosporine, Dasatinib and Sunitinib) the sequence-based kinase distance [5] was plotted against the bioactivity distance for pairs of kinases (shown in Additional file 5: Figure S5). In approximately 57% of the cases, kinases inhibited by the same compound are quite distant according to the sequence-based classification (distance 0.6 – 0.8), but rather close according to the bioactivity-based classification employed in the current work (distance 0 – 0.4). Furthermore, it is known that the gatekeeper residue in kinases is important for determining selectivity against inhibitors [40]. Hence, we also examined whether kinase pairs sharing the same gatekeeper residue were close in bioactivity distance. The results are shown in Additional file 6: Figure S6, where it can be seen that kinase pairs with the same gatekeeper residue occur much more often in the lower pairwise bioactivity distance ranges, than they do in the higher ranges: the occurrence of kinase pairs with the same gatekeeper residue averaged over the first 5 bins (distance range 0 to 0.50) is 23%, whereas it is only 13% for the last 5 bins (distance range > 0.50). These results suggest that the bioactivity-based classification may be more useful globally in predicting kinase inhibitor cross-reactivity than the previous sequence-based classification [5].

### Relationship between SAR similarity and bioactivity distance

Next, the integrity of the kinase tree was examined, which also puts those points into a statistically meaningful context. In this part of the analysis, we attempted to determine to what extent the tree in question was useful for predicting promiscuity of kinase inhibitors; *i.e.* whether kinases which share a similar bioactivity profile and hence are close in bioactivity space are also represented as close neighbors in the tree (and *vice versa*). We hence assessed the number of shared active compounds between each pair of kinases as a measure for SAR similarity and compared this number to the distance based on the bioactivity profiles (shown in Additional file 7: Figure S7). For each kinase, except for NEK7, which was not inhibited by any compound, this pairwise comparison was carried out against all 224 kinases in the dataset.

**Figure 5:**
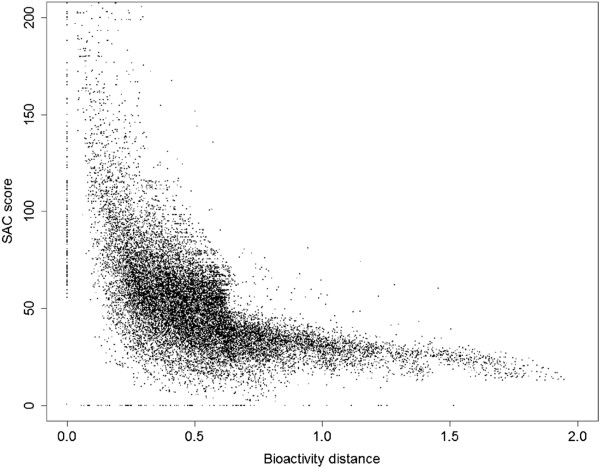
**Mean-centered SAC score versus distance plot.** Mean centering was performed with respect to both axes in order to better visualize the collection of data points: the average distance was set to 0.5 and the average percentage of shared active compounds was set to 50%, and was called ‘SAC score’ after scaling. A clear, negative relationship was observed with most data points (60%) clustered between SAC score ranges of 40 and 100 and distance ranges of 0.2 and 0.6. Extreme SAC score values above 200 were observed for distances smaller than 0.3. Data points with distances larger than 1.0 were less common (only 4%) and compared to the variation in SAC score observed for data points in distance ranges below 0.5 (between SAC score values of 0 and 200), relatively little variation in SAC scores was observed for these data points (between SAC score values of 20 and 40).

Given that a larger distance in the phylogenetic tree indicates less similarity between the kinase pair, a negative relationship between the percentage of shared active compounds and distance of kinases in bioactivity space was expected: In other words, distant kinases are expected to have a relatively low percentage of shared active compounds, whereas neighboring kinases are expected to have a relatively high percentage of shared active compounds. After mean centering of both variables (see Methods section for details) the resulting series are shown in Figure 5, where the percentage of shared active compounds is referred to as ‘SAC score’ (Shared Active Compound score) after mean centering. As expected, a negative relationship was observed between increasing distance in bioactivity space and SAC score, with 60% of the data points clustered between SAC score ranges of 40 and 100 and distance ranges of 0.2 and 0.6. Extreme SAC score values above 200 were observed for distances smaller than 0.3. Data points with distances larger than 1.0 were less common (representing only 4% of the dataset), and compared to the variation in SAC score observed for data points in distance ranges below 0.5 (between SAC scores of 0 and 200), relatively little variation in SAC score was observed for these data points (between SAC scores of 20 and 40). These results suggest that SAR similarity between kinases decreases with higher distance of bioactivity profiles, with changes in the percentage of shared active compounds being the highest for bioactivity profile distances smaller than 0.5.

However, there are a number of factors that deserve attention in this type of analysis: the number of kinases exhibiting a negative relationship between SAC score and bioactivity distance changes drastically, depending on the normalization method used. When the number of shared active compounds was normalized by the total number of active compounds against the common kinase in the pairwise comparison (*i.e.* the kinase which does not change in the pairwise comparisons: when kinase 1 is compared to itself and all other 224 kinases, kinase 1 is referred to as the ‘common’ kinase), the expected negative relationship between SAC score and bioactivity distance (distant kinases having a relatively low percentage of shared active compounds and neighboring kinases having a relatively high percentage of shared active compounds) was only observed in 25% of all kinases (55 out of 224). When the number of shared active compounds was normalized by the total number of compounds active, against both the common kinase and the variable kinase (*i.e.* the other kinase in the comparison), the expected negative relationship was already observed in 46% of all kinases (103 out of 224). When normalized against the total number of compounds active against the *variable* kinase however, the expected relationship was observed in up to 81% of all kinases (181 out of 224). This can likely be explained as follows: when normalizing by the total number of active compounds against the common kinase, all data points in the series are normalized by the same value, and therefore, variable kinases with a high total number of active compounds are more likely to have higher SAC scores (more chances of having shared active compounds), compared to kinases with a lower total number of active compounds. This bias leads to incorrect comparisons, for example in the situation where the variable kinase has a low total number of active compounds, of which a higher percentage is shared with the common kinase, where normalizing by the total number of active compounds against the common kinase would underestimate SAR similarity due to a lower count in shared active compounds (despite a high percentage). Therefore, this percentage, which is the result of normalization by the *total number of compounds active against the variable kinase*, was used in subsequent analyses, also since it was consistent with the SAR trend in the highest number of kinases (81%) included in the analysis. Examples of series showing the expected negative relationship between SAC score and bioactivity distance and series not showing this relationship (kinase outliers) are shown in Additional file 8: Figure S8.

Alternative method of assessing kinase bioactivity distance as described by Bamborough *et al.*[21].

**Figure 6:**
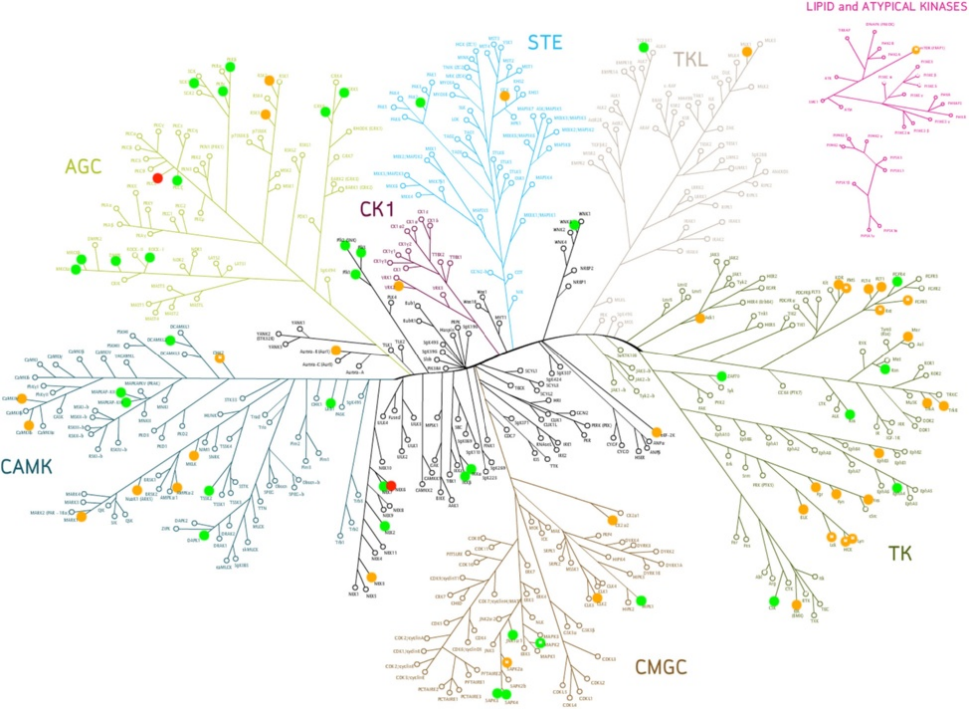
**Kinases that do not show the expected, negative relationship between SAC score and bioactivity distance (kinase outliers).** Kinase outlier group 1 is based on distances generated from fingerprint enrichment profiles (shown in orange) whereas kinase outlier group 2 is based on distances generated from Tanimoto comparison between bioactivity fingerprints of kinases, as performed earlier by Bamborough *et al.* (shown in green) [21]. Whilst the number of outliers is approximately the same for both analyses, they only have 2 kinase outliers in common, NEK6 and KPCI (shown in red). Interestingly, kinases from outlier group 1 share a much higher number of active compounds within the dataset (85.77) as compared to kinases from kinase outlier group 2 (12.03) and few shared activities have been found to be one of the limitations of analyses such as the one performed here (see main text for details). The kinases are distributed in multiple branches, but especially in the TK branch (group 1) and the AGC branch (group 2).

**Figure 7:**
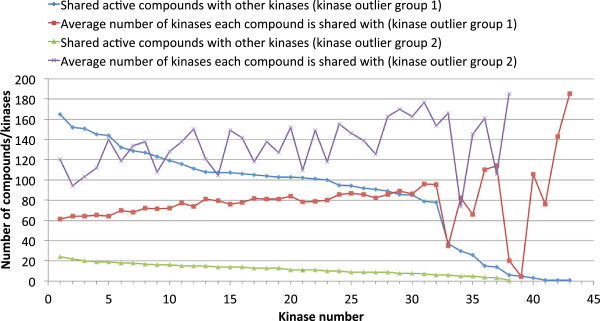
**Compound promiscuity for kinase outlier group 1 and kinase outlier group 2.** Kinase outlier group 1 is based on distances generated from fingerprint enrichment profiles, whereas kinase outlier group 2 is based on distances generated from Tanimoto comparison between bioactivity fingerprints of kinases, as performed earlier by Bamborough *et al.*[21]. Given that the kinases in outlier group 1 share over 7 times as many active compounds with other kinases in the dataset as compared to kinases from outlier group 2, kinase outliers from group 1 have more robust data for SAR similarity comparison and are therefore more likely to be genuine outliers (since their character as outliers is based on more comprehensive underlying data).

In a second calculation, an alternative method of calculating kinase bioactivity distance was employed for comparison, as described by Bamborough *et al.*[21], (see Methods section for details). Using this bioactivity distance, based on Tanimoto comparison between bioactivity fingerprints of kinases (*i.e.* inhibitors were either considered ‘active’ or ‘inactive’ against kinases and chemical features of inhibitors were not taken into account), 185 kinases (83%, so a similar number to the 81% identified above) showed a negative relationship between SAC score and bioactivity distance. Kinase outliers not showing this expected relationship from both analyses are shown in Additional file 9: Table S1 and are highlighted in Figure 6. Whilst the number of outliers is approximately the same for both analyses, they only have 2 outliers in common (namely NEK6 and KPCI). Next, we investigated the outliers in more detail and found that the kinase outliers resulting from the analysis based on fingerprint enrichment profiles (kinase outlier group 1) and those from Tanimoto coefficients on bioactivity profiles (kinase outlier group 2) differ significantly with regard to the distribution of shared bioactivities between kinases: compounds from kinase outlier group 1 share a much higher number of active compounds with other kinases in the dataset (on average 85.77 compounds shared with other kinases), compared to kinase outlier group 2, where on average only 12.03 compounds are shared with other kinases. The distribution of shared activities both in terms of the number of compounds shared, as well as number of kinases the activities are shared with, is shown for the two groups of kinase outliers in Figure 7. Hence, the reasons for both groups of kinases forming outliers is very different: given that the kinases in outlier group 1 share over 7 times as many active compounds with other kinases in the dataset as compared to kinases from outlier group 2, kinase outliers from group 1 have far more robust data for SAR similarity comparison, but they are at the same time much less likely to be placed into a metric space (*i.e.* in a reasonable location with meaningful distances to all other kinases in the dataset). For kinases from outlier group 2 the reason that they form outliers is more likely that there is not sufficient information about their location in ‘bioactivity space’ available in the first place, since their inhibitors are not shared with a sufficient number of other kinases in the dataset.

**Figure 8:**
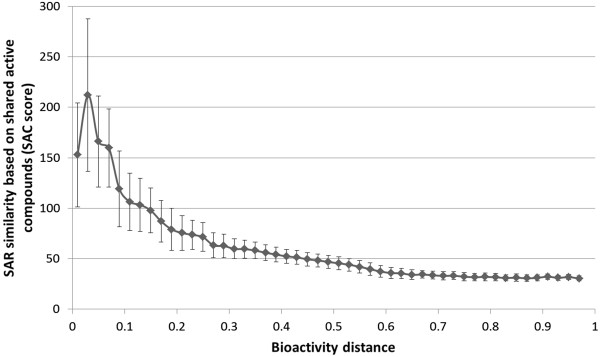
**Average trend of SAR similarity based on shared active compounds for all kinases (181) showing the expected negative relationship between SAR similarity and bioactivity distance (excluding kinases from outlier group 1).** Error bars indicate 1 standard deviation. The number of shared active compounds was normalized by the total number of active compounds in the variable kinase. Interestingly, the optimum in SAR similarity for kinases is not at the lowest distances: kinases show a lower degree of SAR similarity at distances smaller than 0.03 than the optimum which is at a distance of approximately 0.03. Furthermore, variance in shared active compounds decreases with increasing bioactivity distance (after distance is approximately 0.03), suggesting that it is easier to predict SAR similarity for distant kinases than for closely related kinases.

The SAC scores for all 181 kinases which followed the expected relationship between SAC score and bioactivity distance according to our fingerprint enrichment analysis were binned and averaged, the result of which is shown in Figure 8. Interestingly, the highest SAR similarity for kinases is not at the lowest distances: kinases show a lower degree of SAR similarity at distances smaller than 0.03, while the highest SAR similarity is only seen at a distance of approximately 0.03. This observation is most likely an artifact introduced by mean centering of SAC score and distance, but could potentially also be observed as a result of the lack of data points for distance values below 0.03 (as described earlier, the majority of data points (60%) lie outside this range, namely between distance values of 0.2 and 0.6). Thereafter, SAR similarity declines steadily with increasing distance. Another important observation is that also the standard deviations of SAC score values steadily decrease with increasing distance (depicted as error bars). This indicates that there is more variance in kinase SAR similarity for more closely related kinases (*i.e.* kinase pairs with distance < 0.01), than there is for more distant or very distant kinases (*i.e.* distances above this threshold), making prediction of SAR similarity easier for distant kinase pairs. In order to compare our results, we relate our results to previous work based on binding pocket similarity in the following section.

### Comparison to 3D methods

An earlier study by Kuhn *et al.*[32] described a 3D protein binding pocket description and comparison method (Cavbase), which has been utilized to predict kinase inhibitor interaction profiles [41]. In this previous study, the sequence-based similarity of kinases was compared to their Cavbase (*i.e.* three-dimensional similarity) similarity: in many cases kinase pairs exhibit a sequence identity below 50%, while possessing a Cavbase R_1_ similarity score of 22 or above (*i.e.* a high similarity in binding site properties, and hence, a high predicted SAR similarity) [32]. Of the kinase outliers detected in our analysis, Kuhn *et al*. also discovered that the kinases LCK, FGFR1, AKT2, DAPK1 and TGFR1 have unexpected binding site similarities (and hence, unexpected predicted SAR similarities) with sequence-wise distant kinases, which is in accordance with our analysis [32]. In addition, the kinase MK12 (outlier from group 2) also showed low Cavbase predicted SAR similarity against closely related kinases. Similarly, Vieth *et al.* have also shown that the kinases AKT1 (outlier from group 2) and LCK (outlier from group 1) have unexpected SAR similarity with one or more other kinases (which may represent either high SAR similarity despite low sequence similarity, or low SAR similarity despite high sequence similarity) [23]. Our findings show that whilst the majority of kinases (approximately 80% of the kinases in the dataset) exhibit consistent SAR with their neighbors, a subset of kinases does not. Therefore, accurately extrapolating compound activities to these ‘atypical’ kinases, as performed in the study by Martin *et al.*[34], poses an even larger challenge than is generally the case in the area of structure-activity modeling.

### Limitations of phylogenetic clustering of the kinome

Hence, based on the data used in this study, the kinome tree may not be an entirely accurate representation of the information at hand when analyzing and representing chemogenomics relationships between receptors. Both cases with too little data and those that show inconsistent SAR with neighboring kinases are the root of those problems: some kinases show SAR that is similar to other kinases, but not to kinases nearby, and they can thus not be assigned a proper position in a phylogenetic tree. Apart from the problem mentioned earlier – that outliers in bioactivity space can be caused by kinases with insufficient number of shared active compounds (care needs to be taken with respect to data density) – the assumption that kinase SAR can be projected into a metric space represents in our view the second widely used, but still not entirely correct way to represent chemogenomic relationships between targets and their similarities in SAR space. The latter assumption is made by phylogenetic kinome trees and should be reconsidered when conducting chemogenomics analyses.

### Visualization of kinases using multi-dimensional scaling (MDS)

**Figure 9:**
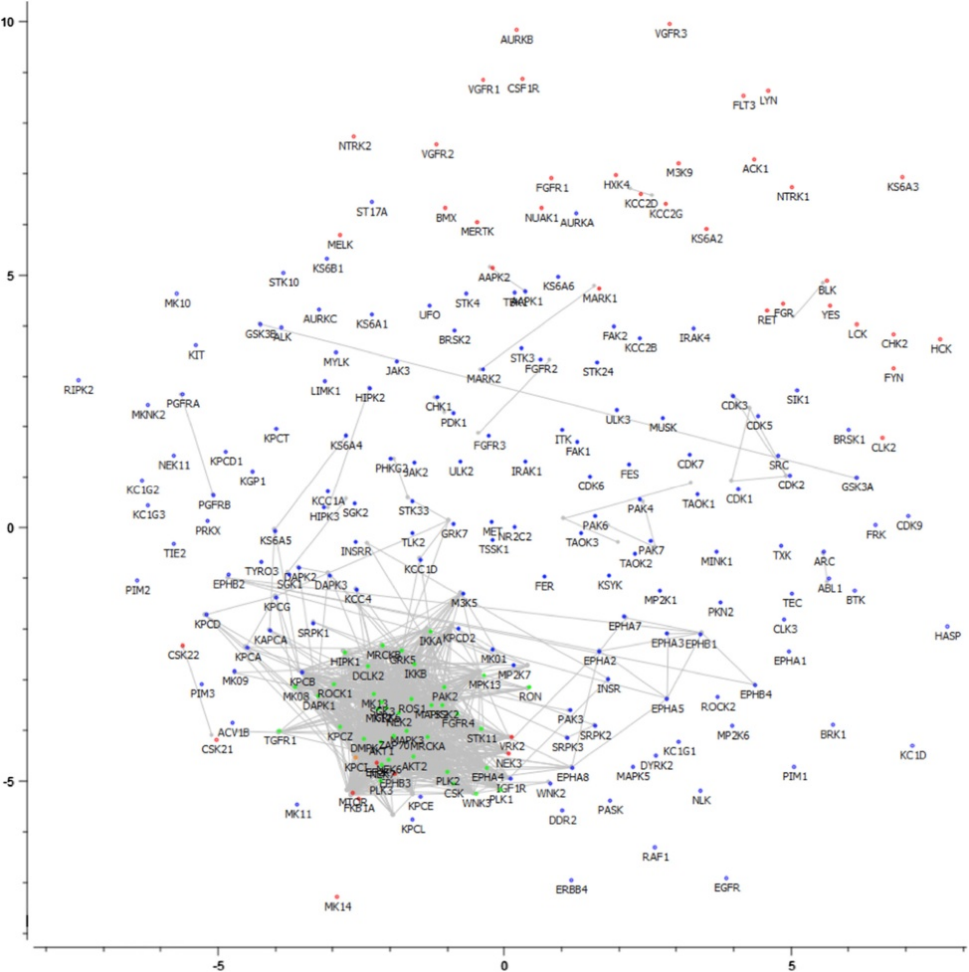
**Multi-dimensional scaling (MDS) of kinases in bioactivity space.** A low average signed relative stress level of 0.28 was obtained, meaning that the 2D representation of the kinases involves a low loss of information. Gray lines connect similar kinases. Kinases in outlier group 1 (shown in red) are clearly separated from the non-outliers, but vary amongst each other in terms of SAR similarity. In contrast, members of the second group of kinase outliers are densely scattered in a small area, indicating that these kinases are very similar to each other in terms of SAR similarity, but are apparently quite distinct from the non-outliers (shown in green). However, it is likely that the kinases in outlier group 2 tend to cluster together, due to the fact that most of these kinases share few active compounds with the other kinases in the dataset, making accurate comparison in terms of SAR similarities more difficult.

In order to alleviate this problem, we next performed multi-dimensional scaling (MDS) of the kinases based on bioactivity fingerprints (shown in Figure 9 and Additional file 10: Figure S9; see Methods section for details). Interestingly, the kinase outliers (as determined by both methods) have 2 distinct distributions. Firstly, kinase outliers resulting from the analysis based on fingerprint enrichment profiles are sparsely distributed and are clearly separated from the non-outlier kinases (group 1 – shown in red); however, kinases within this group are rather dissimilar to each other. Secondly, kinase outliers resulting from the analysis based on the Tanimoto comparison between bioactivity fingerprints of kinases are densely scattered in a small area (group 2 – shown in green). This suggests that kinases in a certain - rather large - area of the kinome (comprising of both group 1 and 2 outliers) space do not show the expected negative relationship between SAC score and bioactivity distance. In contrast to members of the first group, members of the second group of kinase outliers are very similar to each other in terms of bioactivity with an average distance of 0.15 within the group, but quite distinct from the non-outliers (the average distance in the entire dataset is 0.50). However, a closer look at the dataset reveals that the kinases in outlier group 2 *do* tend to cluster together, but simply due to the fact that most of these kinases share few activities with the other kinases in the dataset (less than approximately 12.03 activities compared to 85.77 activities for kinases from outlier group 1, and to on average 51 activities for all kinases in the dataset), making accurate comparison in terms of SAR similarities more difficult. For example, NEK_6 shares only one active compound with other kinases and therefore, can only have either 0% or 100% shared active compounds (before scaling) with other kinases, which introduces unreliable bioactivity relationships in the SAC score-distance plots. Given this finding we repeated the analysis described above for a subset of the original dataset that excluded kinases that had 16 or fewer shared activities (which represents approximately 10% of the maximum number of shared activities for all kinase). The excluded kinases (37 out of 225) are listed in Additional file 11: Table S2.

### Analyses for subset excluding kinases with few shared activities

**Figure 10:**
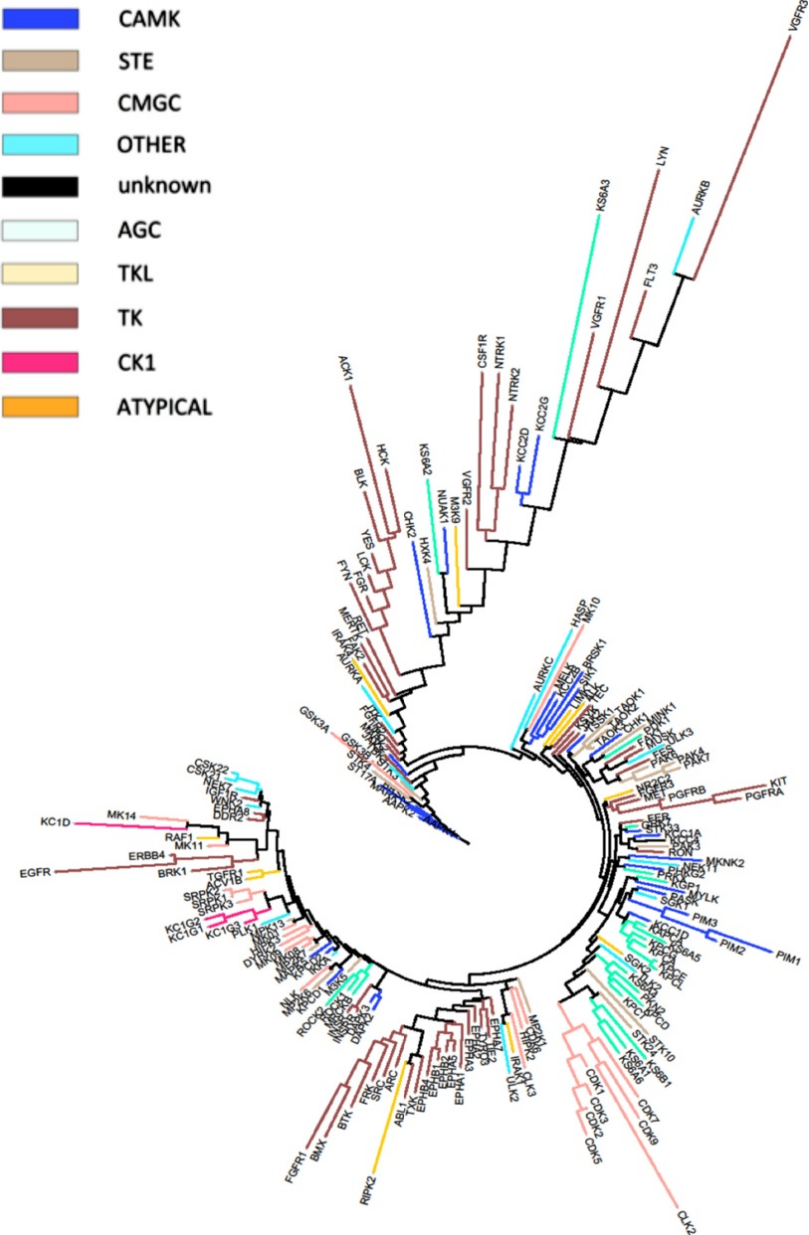
**Kinome tree constructed with a distance matrix based on fingerprint enrichment profiles after exclusion of kinases with 16 or fewer shared activities.** Kinases were colored based on the classification of kinase groups as defined by Manning *et al*[5]. The new tree shows good agreement with the tree constructed earlier. In particular, CDK and CLK kinases are grouped together (with only CDK6 being out of the cluster). Protein C kinases are slightly more spread over 2 small clusters, but as a whole still remain close in the new tree as well. Tyrosine kinases remain clustered together, in particular the Ephrin kinases.

**Figure 11:**
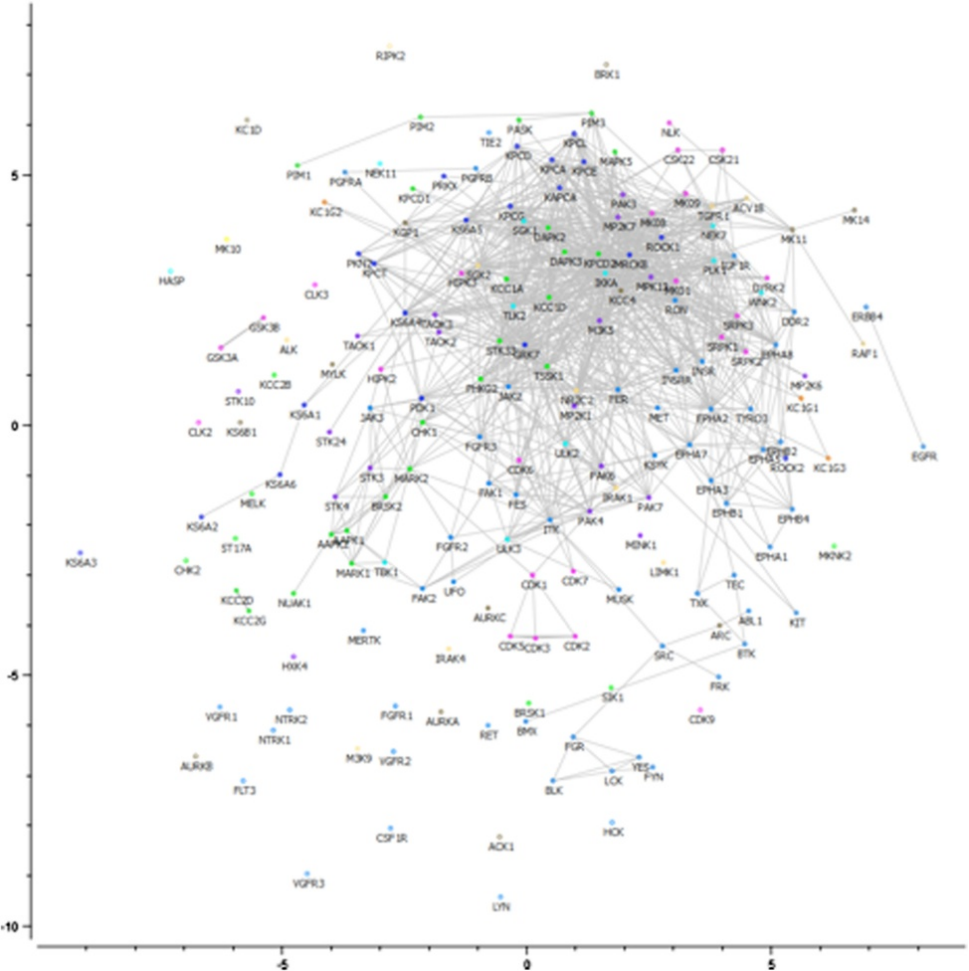
**Multi-dimensional scaling (MDS) of kinases in bioactivity space after omission of kinases with too few shared activities.** Kinases are colored according to their sequence-based classification [5]. A low average signed relative stress level of 0.28 was obtained, meaning that the 2D representation of the kinases involves a low loss of information. Gray lines connect similar kinases. The outliers, based on fingerprint enrichment profiles, are grouped in 2 clusters (indicated by a red line), located far away from each other in bioactivity space, rather than a large one. The kinases VEGFR3, ACK1 and LYN are located far away from the non-outlier kinases, whereas the kinases CSK21, CSK22, IGF1R and WNK2 are located much closer to the non-outliers.

The resulting phylogenetic tree excluding kinases with too few data points is shown in Figure 10, and the corresponding MDS plot based on bioactivity fingerprints is shown in Figure 11. The phylogenetic tree visualized in Figure 10 is more robust than the tree shown earlier, with only 4% of the kinases being outliers (as opposed to ~20% in our earlier analysis). As kinases with too few data points are omitted, this tree therefore significantly improves upon previous analyses that also included rather unreliable data points. However, the overall structure still shows good agreement with that of the tree constructed earlier (as the data for the kinases which were not omitted did not change). In particular, CDK and CLK kinases are grouped together (with only CDK6 being out of the cluster). Isoforms of Protein Kinase C are slightly more spread over 2 small clusters, but as a whole still remain close in the new tree as well. Tyrosine kinases remain clustered together, in particular the Ephrin kinases (which are part of the RTK family of kinases). CAMK kinases, on the other hand, show better clustering in the new tree: only 20% of CAMK kinases were not placed near other CAMK kinases, compared to 31% in the earlier tree. These observations show that exclusion of kinases with few shared activities does not alter the tree drastically: main observations with regards to kinase classification made earlier still apply (but there is still an improvement in clustering). On the other hand, it is known that kinase subtypes (*e.g.* FGFR1 and FGFR2) have similar SAR and tyrosine kinases such as the FGFR, VEGFR, PDGFR and ABL kinases show high cross-reactivity [42]. Still, our revised phylogenetic tree is unable to cluster the 2 groups of kinases mentioned above. In the case of the kinase subtypes this is most likely due to the fact that despite being subtypes of each other, these kinases (FGFR1 and FGFR2) differ across 14% of all bioactivity data points. In the case of the tyrosine kinases it is most likely due to the fact that despite being promiscuous, these kinases still have very different bioactivity profiles.

In our revised analysis, kinases showed much better agreement with respect to the expected relationship in SAC score-distance plots: according to the analysis based on fingerprint enrichment profiles, only 7 kinases (VEGFR3, ACK1, LYN, CSK21, CSK22, IGF1R and WNK2) were classified as outliers (see Methods section for more details on assessment of relationships in SAC score-distance plots), mainly in the tyrosine kinase branch (see Figure 1). Previously, 43 kinases were classified as outliers, of which only 8 were omitted due to lack of shared activities with other kinases in the panel. Hence, based on the data analyzed in this study, our revision of the kinome phylogenetic tree shows that omission of kinases with 16 or less shared activities with other kinases in the panel leads to the construction of a more reliable ligand-based kinome tree, which is more consistent with the observed SAR than previous efforts.

**Figure 12:**
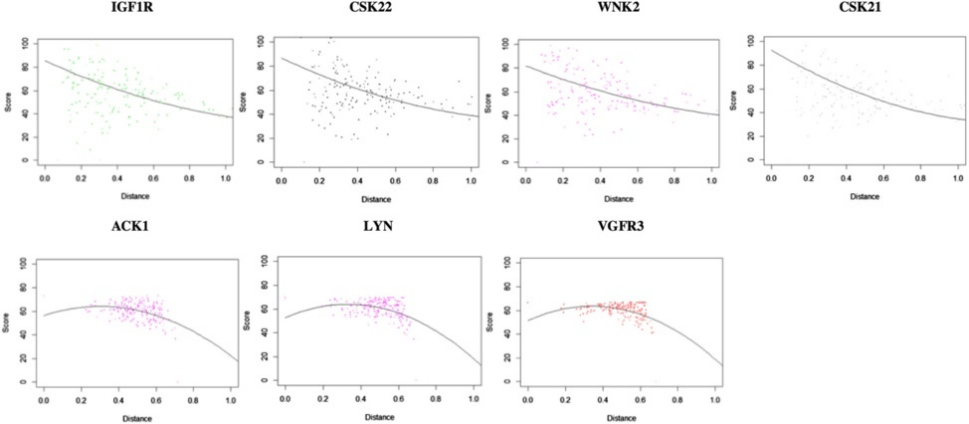
**Two different types of outlier trends, which are likely to explain the formation of 2 clusters of outliers.** VEGFR3, ACK1 and LYN show consistently high SAR similarity with other kinases at both low and high distances, with lower SAR similarity against some kinases at high distances (see graphs below). CSK21, CSK22, IGF1R and WNK2 show significantly higher SAR similarity with other kinases at low distances than at higher distances, but with very high variance of the data points: in many cases, neighboring kinases show low SAR similarity or distant kinases show high SAR similarity (see graphs above). Regardless, 2 different types of outlier trends were observed, possibly explaining the grouping of the outliers in 2 different clusters in the MDS plot.

The MDS plot (Figure 11) shows that the outliers are grouped in 2 clusters, located far away from each other in bioactivity space, rather than in a large one. The kinases VEGFR3, ACK1 and LYN are located far away from the non-outlier kinases, whereas the kinases CSK21, CSK22, IGF1R and WNK2 are located much closer to the non-outliers. After closer inspection of the SAC score-distance relationships of the outlier kinases, we observed 2 different types of outlier trends (see Figure 12), which possibly explain the formation of 2 clusters of outliers. VEGFR3, ACK1 and LYN show consistently high SAR similarity with other kinases at both low and high distances, with lower SAR similarity against some kinases at high distances. On the other hand, CSK21, CSK22, IGF1R and WNK2 show significantly higher SAR similarity with other kinases at low distances than at higher distances, but with very high variance of the data points: in many cases, neighboring kinases show low SAR similarity or distant kinases show high SAR similarity.

Hence, while our removal of kinases with too few data points led to improvement in the reliability of the ligand-based kinome tree produced, there are still structure-activity relationships that are intrinsically difficult to transfer between kinases (bioactivity similarity is after all a local concept [43], not a global one), however on a much smaller scale than before. The main purpose of this work is to draw particular attention to this fact, which is here applied to the chemogenomics analysis of kinase inhibitors, but which is also transferable to other target families. In addition, while it is possible that different assay types (*e.g.* a binding assay instead of a functional assay) may influence the conclusions drawn here, we believe this is unlikely due to the fact that the dataset did not consist of agonists (in which case binding assays and functional assays may not correlate well in every case), but only of antagonists.

## Conclusions

Understanding kinase inhibitor promiscuity still remains a great challenge within the field of drug discovery [44]. In this work, we introduced a revised kinome classification of 225 kinases, based on a complete bioactivity matrix. While kinases from the same group generally tend to arrange in the same cluster, we also observed inconsistencies in the SAR-based kinome trees generated: 80% of all kinases exhibit an expected negative relationship between SAR similarity and bioactivity distance, whilst approximately 20% do not. Two groups of kinase outliers were observed. The first group of outliers resulted from the analysis based on fingerprint enrichment profiles, and show inconsistent SAR similarity to neighboring kinases. The second group of outliers resulted from the analysis based on the Tanimoto comparison between bioactivity fingerprints of kinases, and were observed because these kinases have too few shared activities (data points) to reliably include in the analysis. Exclusion of kinases with a low number of shared activities across the kinase panel (16 or fewer activities) resulted in more robust data with less noise (*i.e.* no kinases with too few data points) and is therefore an improvement on our earlier analysis. This analysis resulted in only 7 out of 188 kinases (4%) being classified as outliers. Interestingly, these outliers were grouped together in 2 clusters in an MDS plot based on bioactivity. Further investigation of their SAR-distance relationships showed that each cluster showed a different relationship between SAR similarity and distance, explaining their MDS classification into 2 groups. Our findings show that while the phylogenetic tree based on bioactivity data shows a good overview of kinases in terms of SAR similarity, it does not explain kinase SAR in all cases (~4% of kinases do not exhibit global SAR similarity to other kinases which share local SAR similarity). Some kinases still need to be repositioned from both the sequence-based kinome tree [5] as well as from previous bioactivity-based kinome classifications, as tree-like structures do not always truly resemble the distance between kinases in SAR space. Hence, based on the data analyzed here, we are able to show that (1) kinases with few shared activities are difficult to establish neighborhood relationships for, and (2) phylogenetic tree representations make implicit assumptions regarding kinase similarities (*i.e.* that neighboring kinases exhibit similar interaction profiles with inhibitors) that are not always suitable for chemogenomics analyses of bioactivity space. These findings are conceptually transferable to other target families.

## Methods

### Dataset

The dataset consists of 157 inhibitors (Calbiochem InhibitorSelect™ Protein Kinase inhibitor library, Cat# 539743-1EA) assayed at concentrations of 1 μM and 10 μM against a panel of 225 kinases (which is publicly available *via* ChEMBL). Bioactivity values are displayed as percentage inhibition, relative to native kinase activity. Compounds that inhibited kinase activity by 50% or more at the concentration screened were considered active. Moreover, same compounds at different concentrations were considered unique for the generation of bioactivity enrichment profiles and the assessment of shared activities (which is elaborated below). Given that all inhibitors were assayed at 2 concentrations, we were able to differentiate between *e.g.* linking kinases by one strong inhibitor and two weak inhibitors. In the former case, features that are present in the inhibitor are counted twice (as the strong inhibitor is active at both concentrations), whereas in the latter case, the features are only present in the active set once per inhibitor (as the weak inhibitors are active only at the higher concentration), and therefore, lead to a different bioactivity profile than in the former case. Using 2 concentrations, we hence emphasize the importance of features present in strong inhibitors.

### Assays

The dataset analyzed was generated at Merck Millipore using the **Kinase**Profiler service [45]. Kinases were diluted in buffers of different composition, depending on the kinase assay, consisting of one or more of the following chemicals: MOPS, EDTA, Brij-35, Glycerol, NaCl, β-mercaptoethanol, BSA, HEPES, Triton X-100, DTT, Triton Surfactant, Glycerol, TRIS, EGTA, Tween 20, Na-β-glycerophosphate and Na_3_VO_4_. Kinase assays involved incubation of the kinase in a buffered solution, followed by the initiation of reaction by addition of an MgATP mix. Reactions were terminated by the addition of a 3% phosphoric acid solution (for some kinases, other solutions were used) after an incubation period (time dependent on kinase) at room temperature. For most kinase assays, 10 μL of the reaction mixes were spotted onto a P30 filtermat and washed thrice for 5 minutes in phosphoric acid (concentration dependent on kinase) and once in methanol prior to drying and scintillation counting [45]. More details on the buffer concentrations used and the specific procedure for each specific kinase assay can be found in the **Kinase**Profiler Service Assay Protocols [45].

### Chemical diversity assessment of inhibitors

MOE version 2011.10 [46] was used to wash and to assign partial charges (the Gasteiger PEOE force field was used) to both the protein kinase inhibitors from ChEMBL (IC_50_ < 10 μM) as well as the inhibitors from Calbiochem InhibitorSelect™ Protein Kinase inhibitor library, Cat# 539743-1EA. Subsequently, principal components of 186 2D molecular descriptors were calculated for all inhibitors.

### Generation of bioactivity-based fingerprint enrichment profiles

Extended connectivity fingerprints with a diameter of 4 bonds (ECFP_4) [47] were used to describe inhibitors, since they were found to capture chemical information correlated with bioactivity in previous studies [48,49]. SMILES string patterns of ECFP_4 features were generated using jCompoundMapper [50]. An active set and an inactive set of compounds (same compounds at different assay concentrations were considered unique) was derived for every kinase with compounds inhibiting kinase activity by 50% or more being considered as active, whilst compounds showing an inhibition of less than 50% being considered as inactive. The enrichment *E*_*i*_ of each *i*^th^ ECFP_4 feature was determined for each kinase by dividing the frequency of the feature in question in the active set of inhibitors (*f*_*A*_) by the frequency in the inactive set (*f*_*I*_):

$$E_{i}=\frac{f_{\text{A}}}{f_{\text{I}}}$$

The Laplacian correction was applied to correct for zero counts in both the nominator and the denominator of the fraction when either of these was equal to zero:

$$E_{\text{i}}=\frac{f_{\text{A}}+1}{f_{\text{I}}+2}$$

This resulted in a bioactivity-based fingerprint enrichment profile for each kinase (kinase vector), referred to as ‘fingerprint enrichment profile’ in the main text. This representation of kinases is somewhat similar to the FragSim similarity measure used by Sutherland *et al.*[51] due to the fact that both measures assess protein similarity by the structures of their inhibitors, but differs in two important aspects. Firstly, the FragSim similarity measure uses larger fragments consisting of 4 to 17 heavy atoms to describe the inhibitors, whereas our fingerprint enrichment profile uses smaller ECFP_4 features. Secondly, the FragSim similarity measure does not take into account the presence of its fragments in the inactive set of compounds, hereby not distinguishing between features which are present only in the active set of inhibitors and features which are present in both the active set as well as the inactive set of inhibitors. This is taken into account in our ‘fingerprint enrichment profile’.

### Generation of distance matrices and kinase inhibitor response-distance relationships

Two types of distance matrices were used for analysis. Firstly, and novel to this work, a distance matrix was constructed based on the fingerprint enrichment profile. The Manhattan distance was calculated between each kinase vector and was normalized by the number of dimensions (*i.e.* features) in the vector, which were obtained using feature counts. Secondly, as shown earlier by Bamborough *et al.*[21], each kinase was represented as a bit-string and each bit represented the activity of a compound (either ‘0’ for inactive compounds or ‘1’ for active compounds). The Tanimoto coefficient was used to assess distances between kinases based on the bioactivity fingerprints. As described in Bamborough *et al.*[21], the distance *D* was calculated from the Tanimoto coefficient *T*_*C*_ as follows:

$$D=\sqrt{\left(1-T_{C}\right)}$$

Each kinase was compared pairwise against all other kinases using both of the above measures. The percentage of shared active compounds was normalized by the total number of active compounds in either the ‘common’ kinase (*i.e.* the kinase which does not change in the pairwise comparisons: when kinase 1 is compared to itself and all other 224 kinases, kinase 1 is referred to as the ‘common’ kinase), the ‘variable’ kinase (*i.e.* the other kinase in the comparison) or in both the kinases. The normalized values were converted to percentages and were plotted against the distance, resulting in a trend series for every kinase. In order to better visualize the collection of data points, mean centering was performed on the series with respect to each axis: the average distance was set to 0.5 and the average percentage was set to 50% and was called ‘SAC score’ after mean centering.

### Assessment of sequence-based similarity distance-bioactivity distance plots

The sequence-based kinase distance matrix was calculated using T-Rex [52] from the tree file obtained from the human kinome project [53]. Kinase pairs targeted by the inhibitor were automatically extracted from the supplementary material provided by Karaman *et al.*[30] and looked up in the sequence-based distance matrix [5].

### Kinase gatekeeper analysis

The kinase gatekeepers were determined by performing a multiple sequence alignment on the kinases using MEGA version 5 [54], using the default parameters (Protein Weight Matrix: Gonnet, Gap Open penalty: 10, Gap Extension penalty: 0.20 and Gap Distances: 5). Subsequently, bioactivity distance between kinases pairs was compared to their gatekeeper residues.

### Generation of phylogenetic trees

PHYLIP was used to create tree files from the distance matrix using the neighbor-joining method (no outgroup root was specified) [55] and Archaeopteryx and iTOL were used for visualization [56-58]. In addition, the Merck Millipore DART tool [59] was used to visualize the sequence-based kinome tree as defined by Manning *et al.*[5] Kinases were colored based on the classification of kinase groups as defined by the sequence-based tree [5].

### Assessment of relationship between SAC score and bioactivity distance

In order to assess the relationship of the 224 SAC score-distance, a second degree polynomial function was fitted through the data points of each series using R [60]. Series with a negative slope at distance = 0.40 and distance = 0.67 (with the highest distance being approximately 1.9), and an R^2^ value greater than 0.2 were considered to be exhibiting neighborhood behavior.

### Generation of multidimensional-scaling (MDS) plots

The Hamming distance was calculated between kinases based on their binary bioactivity fingerprints (compounds inhibiting kinase activity by 50% or more were considered active and compounds inhibiting kinase activity by less than 50% were considered inactive) and an MDS plot was generated using Orange Canvas [61]. Signed relative stress levels were minimized (with stopping conditions being a minimum stress change of 0.00005 and a maximum number of steps of 5000) and kinases were colored either according to their group as determined by sequence-based classification [5] (for Figure 11) or by class (*i.e.* outlier group 1, outlier group 2 or non-outlier – for Figure 9 and Additional file 8: Figure S8).

## Abbreviations

ABL: Abelson murine leukemia viral oncogene homologue 1; ACK1: Tyrosine kinase non-receptor protein 2; AKT1: V-akt murine thymoma viral oncogene homologue 1; AKT2: V-akt murine thymoma viral oncogene homologue 2; ATP: Adenosine triphosphate; BCR: Breakpoint cluster region protein; CDK: Cyclin-dependent kinase; CDK6: Cell division protein kinase 6; CLK: CDC-like kinase 1; CML: Chronic myelogenous leukemia; CSK21: Casein kinase II subunit alpha; CSK22: Casein kinase II subunit alpha’; DAPK1: Death-associated protein kinase 1; ECFP: Extended connectivity fingerprints; FGFR1: Fibroblast growth factor receptor 1; IGF1R: Insulin-like growth factor 1 receptor; KIT: Mast/stem cell growth factor receptor; KPCA: Protein kinase C alpha type; KPCB: Protein kinase C beta type; KPCI: Protein kinase C iota type; LCK: Lymphocyte cell-specific protein-tyrosine kinase; LYN: V-yes-1 Yamaguchi sarcoma viral related oncogene homolog; MDS: Multi-dimensional scaling; MK11: Mitogen-activated protein kinase p38 beta; MK12: Mitogen-activated protein kinase p38 gamma; MK13: Mitogen-activated protein kinase p38 delta; MK14: Mitogen-activated protein kinase p38 alpha; NEK6: Never in mitosis A-related kinase 6; NEK7: Never in mitosis A-related kinase 7; QSAR: Quantitative structure-activity relationship; SAR: Structure-activity relationship; SMILE: Simplified molecular-input line-entry; SRC: Proto-oncogene tyrosine-protein kinase Src; TC: Tanimoto coefficient; TGFR1: Transforming growth factor-beta receptor type I; VEGFR3: Vascular endothelial growth factor receptor 3; WNK2: Serologically defined colon cancer antigen 43.

## Competing interests

The authors declare that they have no competing interests.

## Authors’ contributions

SP carried out the cheminformatics analyses and drafted the manuscript. TK, MA and UAP were involved with the generation of the experimental data and AB devised the cheminformatics analysis of the experimental data. All authors read and approved the final manuscript.

## Supplementary Material

Additional file 1: Figure S1 Chemical diversity assessment of the Calbiochem inhibitor library employed in the current work relative to ChEMBL protein kinase inhibitors with IC_50_ < 10 μM. PC1 (principal component 1) and PC2 (principal component 2) capture 46% of all variance in the dataset and are related to molecular size (PC1) and charge and lipophilicity (PC2). While this dataset hence does not represent all kinase inhibitor space, it places more of an emphasis on the usually more desired smaller compounds, such as Docetaxel (MW of 808 Da). Click here for file

Additional file 2: Figure S2 Frequency of the 10 most frequent scaffolds present in the dataset used. Given the presence of 110 different scaffolds in the dataset, we consider this dataset to be chemically diverse. Click here for file

Additional file 3: Figure S3 Heat map for the dataset employed here comprising 225 kinases assayed against 157 inhibitors at concentrations of 1 μM and 10 μM. Gray dots indicate potent kinase-inhibitor interaction (*i.e.* active compounds), with darker shades of gray corresponding to stronger kinase-inhibitor interaction. 16.1% of all inhibitor-kinase pairs in the data matrix show 50% or more inhibition (which were defined as ‘active’ in the current study). Click here for file

Additional file 4: Figure S4 Distribution of compound-target interactions in the dataset. Of all data present in the dataset, 16.1% of all compound-target interactions represent inhibition by at least 50% and only 2% represent inhibition between 40% and 60%. Click here for file

Additional file 5: Figure S5 Sequence-based [5] distance-bioactivity distance plots for kinase pairs targeted by known inhibitors SU-14813, Gefitinib, Staurosporine, BIRB-796, Dasatinib and Sunitinib. Inhibitor data was acquired from Karaman *et al.*[30]. In most cases, it is clear that there is a big cluster of data points on the left side of the y = x partition, meaning that kinases inhibited by the same compound are quite distant according to the sequence-based classification (distance 0.6 – 0.8), but rather close according to our fingerprint enrichment-based classification (distance 0 – 0.4). These results suggest that the fingerprint enrichment-based classification ismore useful in predicting kinase inhibitor cross-reactivity than the sequence-based classification by Manning *et al.*[5]. Click here for file

Additional file 6: Figure S6 Comparison of gatekeeper residue similarity with bioactivity distance. Kinase pairs with the same gatekeeper residue occur much more often in the lower pairwise bioactivity distance ranges, than they do in the higher ranges, with the occurrence of kinase pairs with the same gatekeeper residue averaged over the first 5 bins (distance range 0 to 0.50) being 23%, whereas it is only 13% for the last 5 bins (distance range > 0.50). Click here for file

Additional file 7: Figure S7 Non-scaled percentage activity versus distance plot for all (224) kinases. Prior to normalization, the raw data is very difficult to interpret. Click here for file

Additional file 8: Figure S8 Examples of kinase SAC score-distance series. The upper two series (kinases KPCD and PIM1) show a negative relationship between SAC score and bioactivity distance, whereas the lower 2 series (kinases MK14 and ACK1) do not. Click here for file

Additional file 9: Table S1 Kinase outliers not showing the expected negative relationship between SAC score and bioactivity distance according to preliminary analysis. Outlier group 1 consists of 43 kinases and outlier group 2 consist of 39 kinases. Both groups only have 2 kinase outliers in common (NEK6 and KPCI). Click here for file

Additional file 10: Figure S9 MDS of kinases, zoomed in on outlier group 2. Kinases from this group show high similarity to each other. However, this apparent similarity is most likely due to the absence of information (*i.e.* shared active compounds), and therefore does not represent true similarity. Click here for file

Additional file 11: Table S2 Kinases with 16 or fewer shared activities with other kinases in the panel. These kinases were excluded from the dataset after a preliminary analysis showed that they had too few shared activities to be able to compare SAR similarities of kinases accurately. Click here for file
