# Supplementary figures and images for: Are phylogenetic trees suitable for chemogenomics analyses of bioactivity data sets: the importance of shared active compounds and choosing a suitable data embedding method, as exemplified on Kinases
Source: J Cheminform. 2013 Dec 13;5:49. doi: 10.1186/1758-2946-5-49 (PMC3900467; doi:10.1186/1758-2946-5-49)

Frequency

0.035  
0.03  
0.025  
0.02  
0.015  
0.01  
0.005  
0

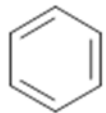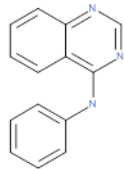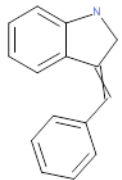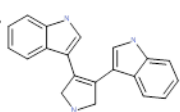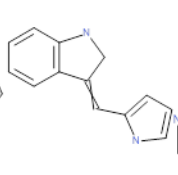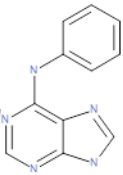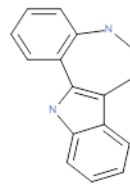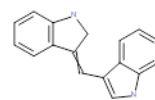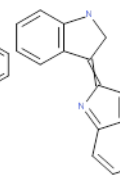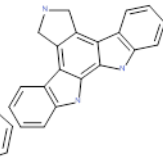

Supplement: Additional file 2: Figure S2 — Frequency of the 10 most frequent scaffolds present in the dataset used. Given the presence of 110 different scaffolds in the dataset, we consider this dataset to be chemically diverse. [file 1758-2946-5-49-S2.pdf]

Kinases →

Compounds ↓

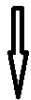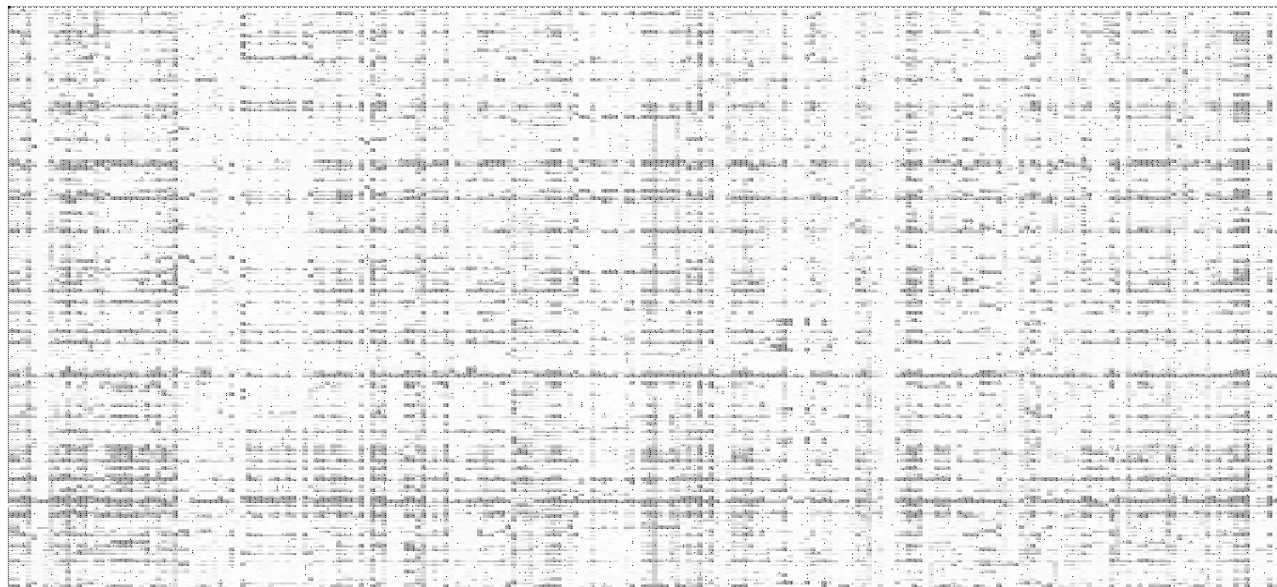

Supplement: Additional file 3: Figure S3 — Heat map for the dataset employed here comprising 225 kinases assayed against 157 inhibitors at concentrations of 1 μM and 10 μM. Gray dots indicate potent kinase-inhibitor interaction (i.e. active compounds), with darker shades of gray corresponding to stronger kinase-inhibitor interaction. 16.1% of all inhibitor-kinase pairs in the data matrix show 50% or more inhibition (which were defined as ‘active’ in the current study). [file 1758-2946-5-49-S3.pdf]

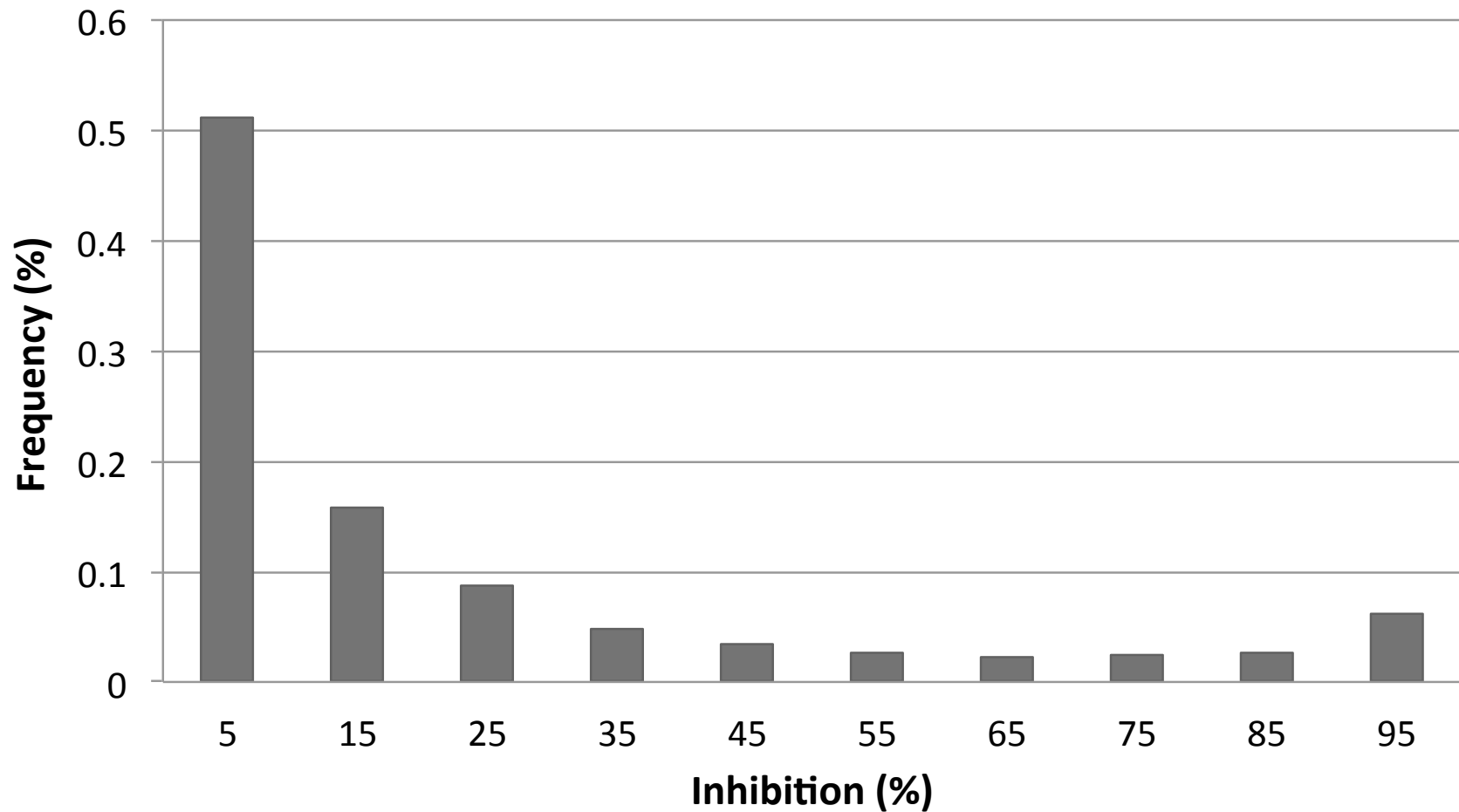

Supplement: Additional file 4: Figure S4 — Distribution of compound-target interactions in the dataset. Of all data present in the dataset, 16.1% of all compound-target interactions represent inhibition by at least 50% and only 2% represent inhibition between 40% and 60%. [file 1758-2946-5-49-S4.pdf]

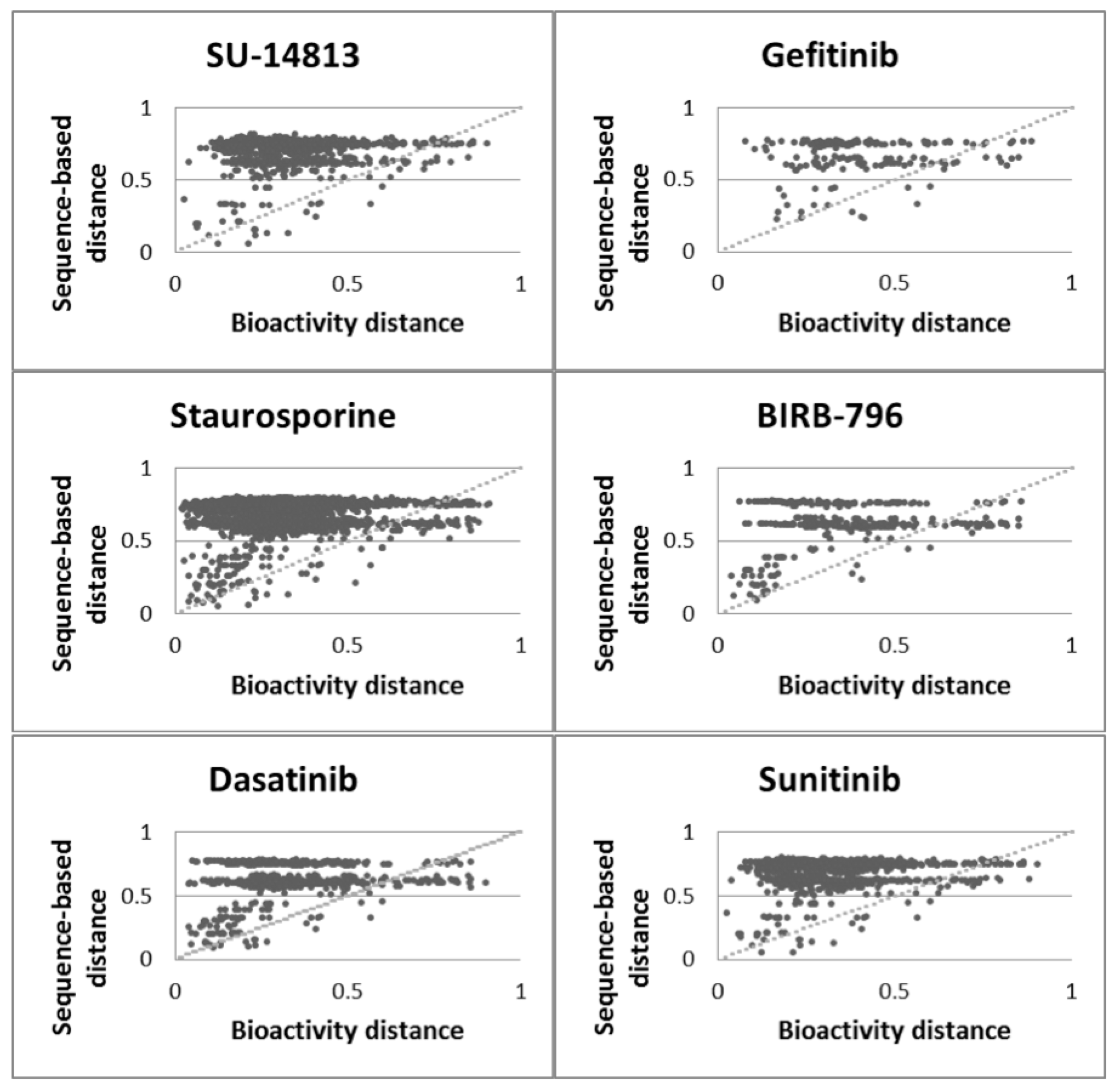

Supplement: Additional file 5: Figure S5 — Sequence-based [5] distance-bioactivity distance plots for kinase pairs targeted by known inhibitors SU-14813, Gefitinib, Staurosporine, BIRB-796, Dasatinib and Sunitinib. Inhibitor data was acquired from Karaman et al.[30]. In most cases, it is clear that there is a big cluster of data points on the left side of the y = x partition, meaning that kinases inhibited by the same compound are quite distant according to the sequence-based classification (distance 0.6 – 0.8), but rather close according to our fingerprint enrichment-based classification (distance 0 – 0.4). These results suggest that the fingerprint enrichment-based classification ismore useful in predicting kinase inhibitor cross-reactivity than the sequence-based classification by Manning et al.[5]. [file 1758-2946-5-49-S5.png]

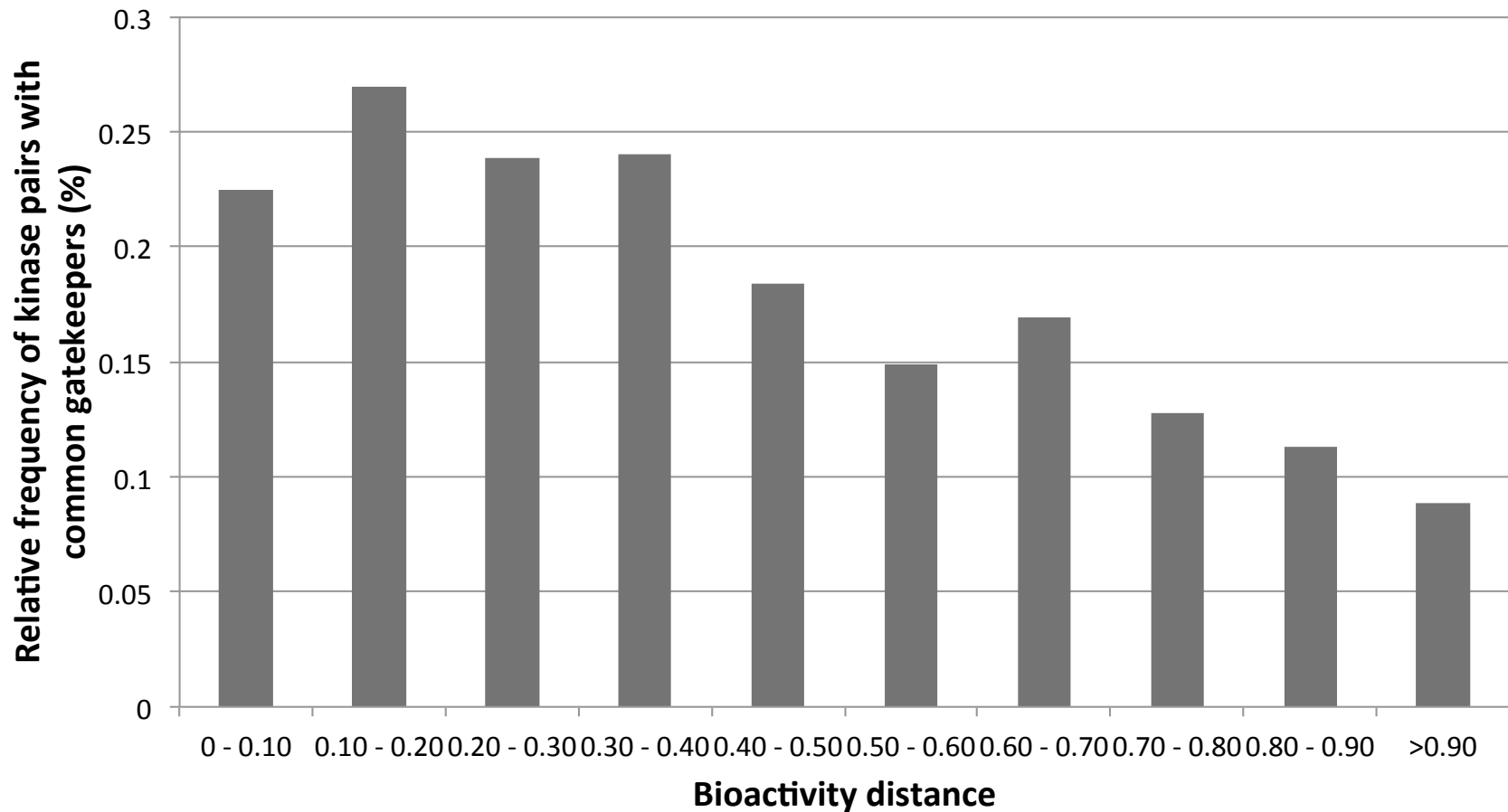

Supplement: Additional file 6: Figure S6 — Comparison of gatekeeper residue similarity with bioactivity distance. Kinase pairs with the same gatekeeper residue occur much more often in the lower pairwise bioactivity distance ranges, than they do in the higher ranges, with the occurrence of kinase pairs with the same gatekeeper residue averaged over the first 5 bins (distance range 0 to 0.50) being 23%, whereas it is only 13% for the last 5 bins (distance range > 0.50). [file 1758-2946-5-49-S6.pdf]

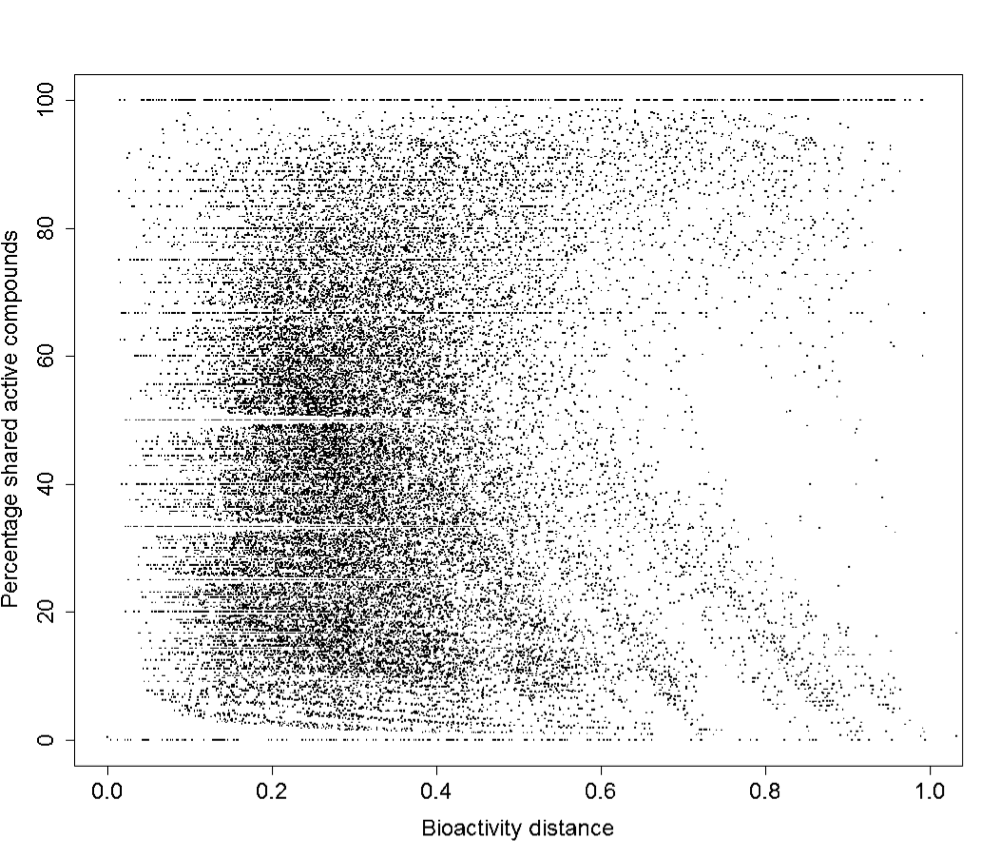

Supplement: Additional file 7: Figure S7 — Non-scaled percentage activity versus distance plot for all (224) kinases. Prior to normalization, the raw data is very difficult to interpret. [file 1758-2946-5-49-S7.png]

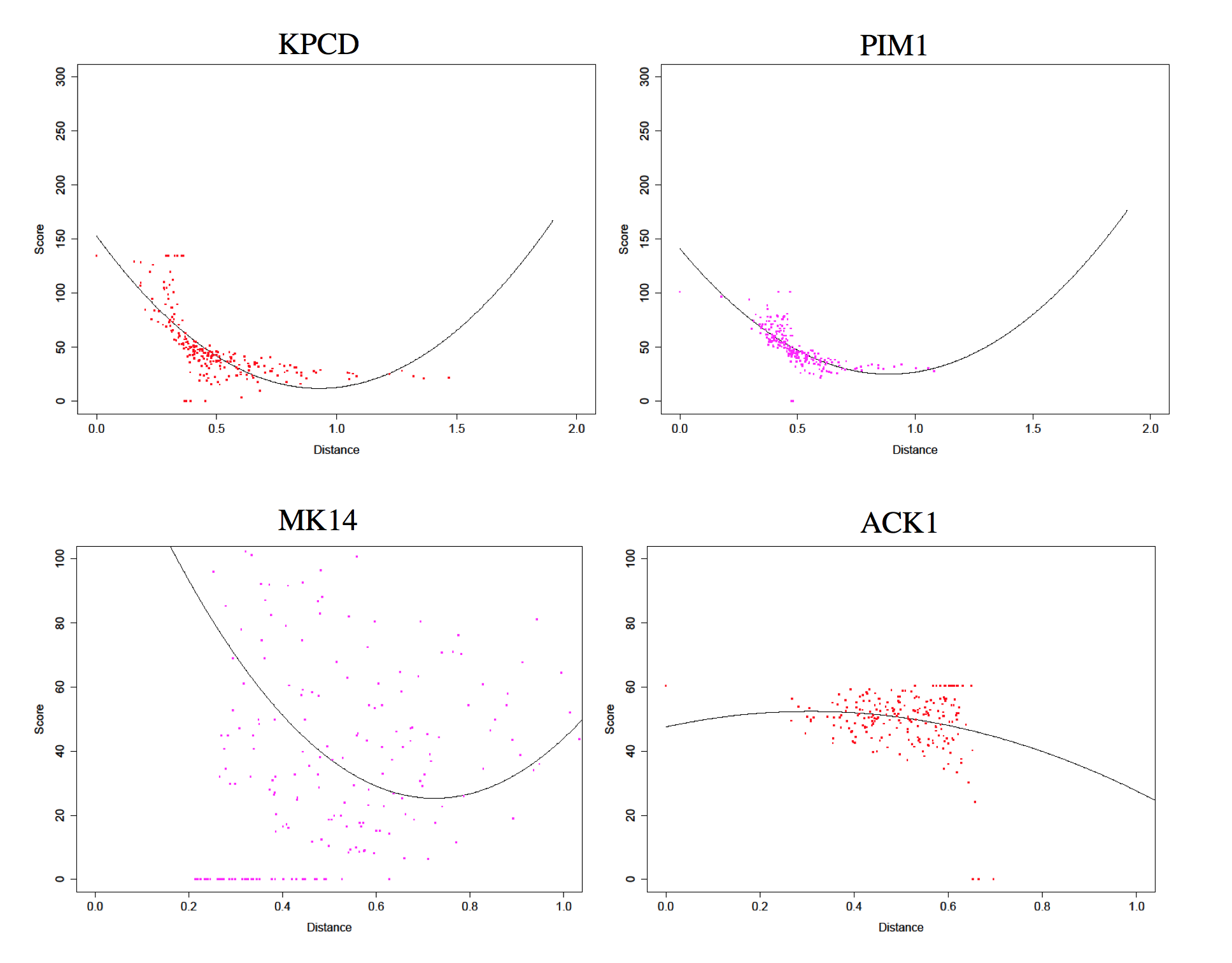

Supplement: Additional file 8: Figure S8 — Examples of kinase SAC score-distance series. The upper two series (kinases KPCD and PIM1) show a negative relationship between SAC score and bioactivity distance, whereas the lower 2 series (kinases MK14 and ACK1) do not. [file 1758-2946-5-49-S8.png]

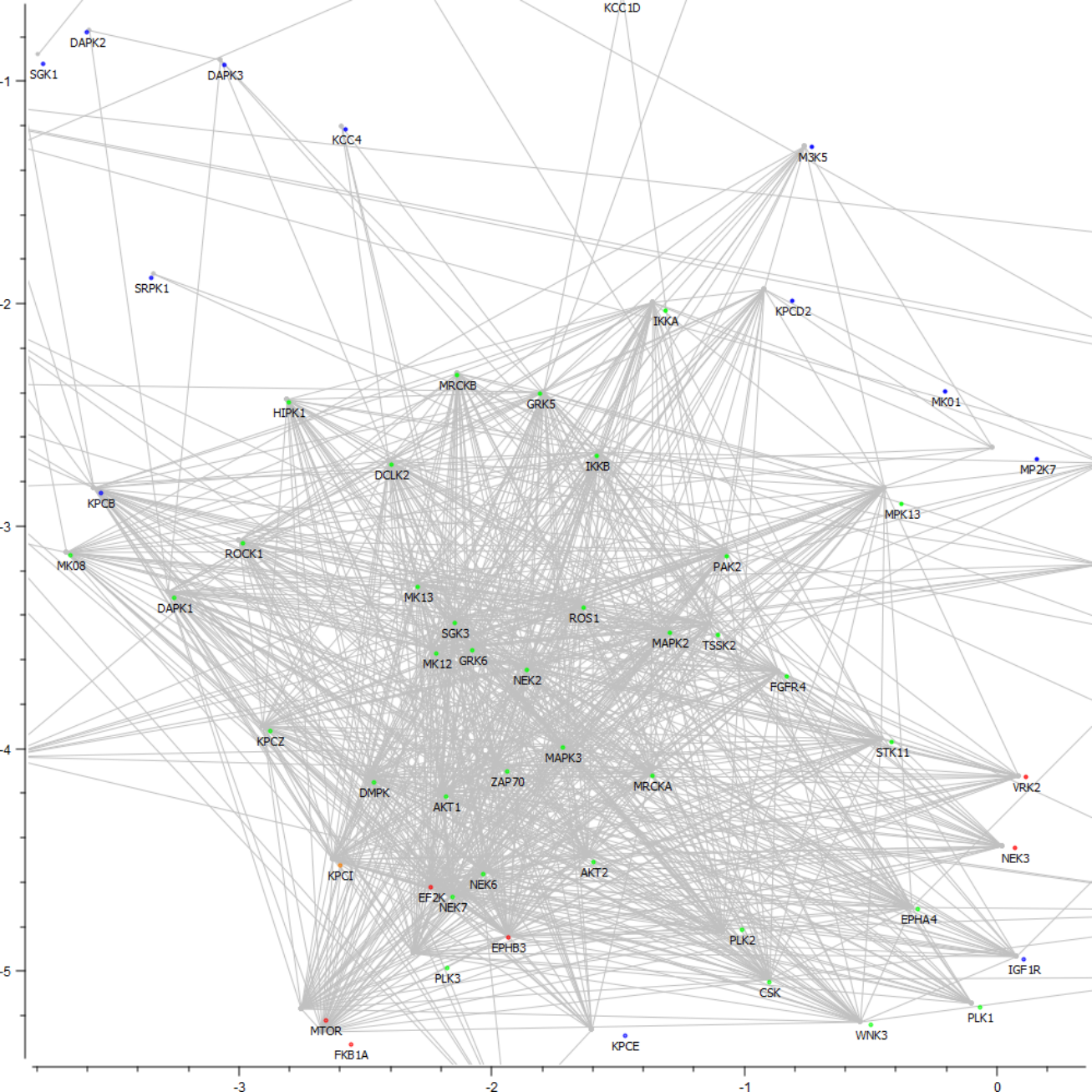

Supplement: Additional file 10: Figure S9 — MDS of kinases, zoomed in on outlier group 2. Kinases from this group show high similarity to each other. However, this apparent similarity is most likely due to the absence of information (i.e. shared active compounds), and therefore does not represent true similarity. [file 1758-2946-5-49-S10.pdf]
