# Supplementary material for: Are phylogenetic trees suitable for chemogenomics analyses of bioactivity data sets: the importance of shared active compounds and choosing a suitable data embedding method, as exemplified on Kinases
Source: J Cheminform. 2013 Dec 13;5:49. doi: 10.1186/1758-2946-5-49 (PMC3900467; doi:10.1186/1758-2946-5-49)
Supplement: Additional file 9: Table S1 — Kinase outliers not showing the expected negative relationship between SAC score and bioactivity distance according to preliminary analysis. Outlier group 1 consists of 43 kinases and outlier group 2 consist of 39 kinases. Both groups only have 2 kinase outliers in common (NEK6 and KPCI). [file 1758-2946-5-49-S9.doc]

Supplementary Table 1. Kinase outliers not showing the expected negative relationship between SAC score and bioactivity distance according to preliminary analysis. Outlier group 1 consists of 43 kinases and outlier group 2 consist of 39 kinases. Both groups only have 2 kinase outliers in common (NEK6 and KPCI).

| **Kinase outlier group 1 (based on distances generated from fingerprint enrichment profiles)** | **Kinase outlier group 2 (based on distances generated from Tanimoto comparison between bioactivity fingerprints)** |
| --- | --- |
| AAPK2 | AKT1 |
| ACK1 | AKT2 |
| AURKB | CSK |
| BLK | DAPK1 |
| BMX | DCLK2 |
| CHK2 | DMPK |
| CLK2 | EPHA4 |
| CSF1R | FGFR4 |
| CSK21 | GRK5 |
| CSK22 | GRK6 |
| EF2K | HIPK1 |
| EPHB3 | IKKA |
| FGFR1 | IKKB |
| FGR | KPCI |
| FKB1A | KPCZ |
| FLT3 | MAPK2 |
| FYN | MAPK3 |
| HCK | MK08 |
| HXK4 | MK12 |
| KCC2D | MK13 |
| KCC2G | MPK13 |
| KPCI | MRCKA |
| KS6A2 | MRCKB |
| KS6A3 | NEK2 |
| LCK | NEK6 |
| LYN | NEK7 |
| M3K9 | PAK2 |
| MARK1 | PLK1 |
| MELK | PLK2 |
| MERTK | PLK3 |
| MK14 | ROCK1 |
| MTOR | RON |
| NEK3 | ROS1 |
| NEK6 | SGK3 |
| NTRK1 | STK11 |
| NTRK2 | TGFR1 |
| NUAK1 | TSSK2 |
| RET | WNK3 |
| VEGFR1 | ZAP70 |
| VEGFR2 |  |
| VEGFR3 |  |
| VRK2 |  |
| YES |  |
