# Supplementary material for: Are phylogenetic trees suitable for chemogenomics analyses of bioactivity data sets: the importance of shared active compounds and choosing a suitable data embedding method, as exemplified on Kinases
Source: J Cheminform. 2013 Dec 13;5:49. doi: 10.1186/1758-2946-5-49 (PMC3900467; doi:10.1186/1758-2946-5-49)
Supplement: Additional file 11: Table S2 — Kinases with 16 or fewer shared activities with other kinases in the panel. These kinases were excluded from the dataset after a preliminary analysis showed that they had too few shared activities to be able to compare SAR similarities of kinases accurately. [file 1758-2946-5-49-S11.doc]

Supplementary Table 2. Kinases with 16 or fewer shared activities with other kinases in the panel. These kinases were excluded from the dataset after a preliminary analysis showed that they had too few shared activities to be able to compare SAR similarities of kinases accurately.

| **Excluded kinases** |
| --- |
| HIPK1 |
| KPCB |
| PLK2 |
| DCLK2 |
| DAPK1 |
| GRK5 |
| NEK3 |
| IKKB |
| PAK2 |
| WNK3 |
| VRK2 |
| EPHA4 |
| TSSK2 |
| STK11 |
| FGFR4 |
| CSK |
| MAPK2 |
| MK13 |
| AKT2 |
| MRCKA |
| NEK2 |
| KPCZ |
| ROS1 |
| GRK6 |
| SGK3 |
| MK12 |
| FKB1A |
| ZAP70 |
| MAPK3 |
| PLK3 |
| DMPK |
| MTOR |
| AKT1 |
| KPCI |
| EF2K |
| EPHB3 |
| NEK6 |
