# Supplementary material for: Are phylogenetic trees suitable for chemogenomics analyses of bioactivity data sets: the importance of shared active compounds and choosing a suitable data embedding method, as exemplified on Kinases
Source: J Cheminform. 2013 Dec 13;5:49. doi: 10.1186/1758-2946-5-49 (PMC3900467; doi:10.1186/1758-2946-5-49)
Supplement: Additional file 1: Figure S1 — Chemical diversity assessment of the Calbiochem inhibitor library employed in the current work relative to ChEMBL protein kinase inhibitors with IC50 < 10 μM. PC1 (principal component 1) and PC2 (principal component 2) capture 46% of all variance in the dataset and are related to molecular size (PC1) and charge and lipophilicity (PC2). While this dataset hence does not represent all kinase inhibitor space, it places more of an emphasis on the usually more desired smaller compounds, such as Docetaxel (MW of 808 Da). [file 1758-2946-5-49-S1.pdf]

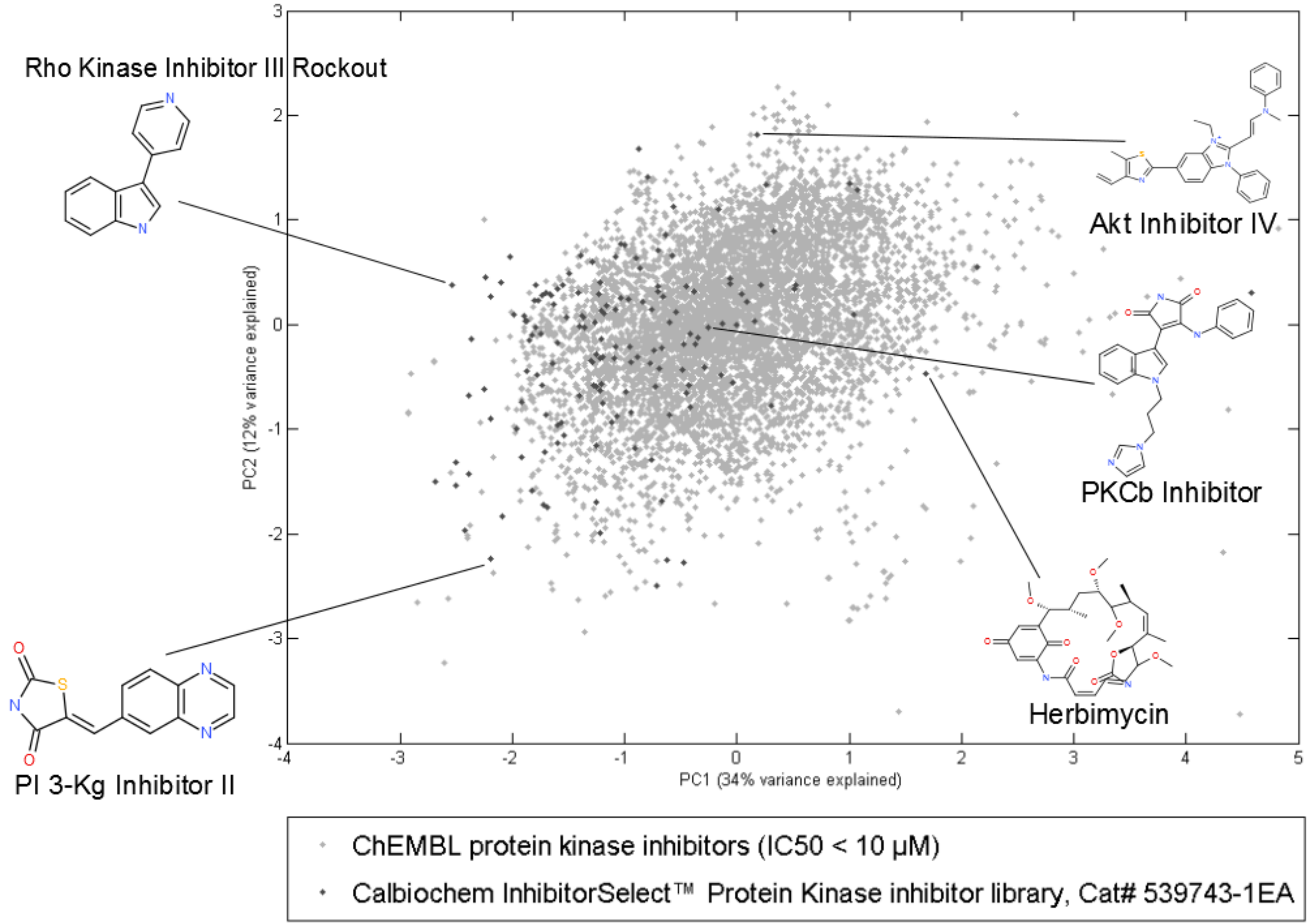

Rho Kinase Inhibitor III Rockout
Akt Inhibitor IV
PKCb Inhibitor
Herbimycin
PI 3-Kg Inhibitor II
PC2 (12% variance explained)
PC1 (34% variance explained)
ChEMBL protein kinase inhibitors (IC50 < 10 µM)
Calbiochem InhibitorSelect™ Protein Kinase inhibitor library, Cat# 539743-1EA
